# Evolution of myeloid-mediated immunotherapy resistance in prostate cancer

**DOI:** 10.1038/s41586-024-08290-3

**Published:** 2024-12-04

**Authors:** Aram Lyu, Zenghua Fan, Matthew Clark, Averey Lea, Diamond Luong, Ali Setayesh, Alec Starzinski, Rachel Wolters, Marcel Arias-Badia, Kate Allaire, Kai Wu, Vibha Gurunathan, Laura Valderrábano, Xiao X. Wei, Richard A. Miller, Eliezer M. Van Allen, Lawrence Fong

**Affiliations:** 1 Division of Hematology/Oncology, Department of Medicine, University of California, San Francisco, San Francisco, CA USA; 2 Parker Institute for Cancer Immunotherapy, San Francisco, CA USA; 3 Immunotherapy Integrated Research Center, Division of Translational Science and Therapeutics, Fred Hutchinson Cancer Center, Seattle, WA USA; 4 Department of Medical Oncology, Dana-Farber Cancer Institute, Boston, MA USA; 5 Broad Institute of MIT and Harvard, Cambridge, MA USA; 6 Corvus Pharmaceuticals, Burlingame, CA USA; 7 Helen Diller Family Comprehensive Cancer Center, University of California, San Francisco, San Francisco, CA USA

**Keywords:** Immunotherapy, Cancer microenvironment

## Abstract

Patients with advanced metastatic castration-resistant prostate cancer (mCRPC) are refractory to immune checkpoint inhibitors (ICIs)^1,2^, partly because there are immunosuppressive myeloid cells in tumours^3,4^. However, the heterogeneity of myeloid cells has made them difficult to target, making blockade of the colony stimulating factor-1 receptor (CSF1R) clinically ineffective. Here we use single-cell profiling on patient biopsies across the disease continuum and find that a distinct population of tumour-associated macrophages with elevated levels of *SPP1* transcripts (*SPP1*^hi^-TAMs) becomes enriched with the progression of prostate cancer to mCRPC. In syngeneic mouse modelling, an analogous macrophage population suppresses CD8^+^ T cell activity in vitro and promotes ICI resistance in vivo. Furthermore, *Spp1*^hi^-TAMs are not responsive to anti-CSF1R antibody treatment. Pathway analysis identifies adenosine signalling as a potential mechanism for *SPP1*^hi^-TAM-mediated immunotherapeutic resistance. Indeed, pharmacological inhibition of adenosine A2A receptors (A2ARs) significantly reverses *Spp1*^hi^-TAM-mediated immunosuppression in CD8^+^ T cells in vitro and enhances CRPC responsiveness to programmed cell death protein 1 (PD-1) blockade in vivo. Consistent with preclinical results, inhibition of A2ARs using ciforadenant in combination with programmed death 1 ligand 1 (PD-L1) blockade using atezolizumab induces clinical responses in patients with mCRPC. Moreover, inhibiting A2ARs results in a significant decrease in *SPP1*^hi^-TAM abundance in CRPC, indicating that this pathway is involved in both induction and downstream immunosuppression. Collectively, these findings establish *SPP1*^hi^-TAMs as key mediators of ICI resistance in mCRPC through adenosine signalling, emphasizing their importance as both a therapeutic target and a potential biomarker for predicting treatment efficacy.

## Abstract (web summary)

Single-cell profiling of human prostate cancer and studies in mouse models show that macrophages expressing *SPP1* mediate immunotherapeutic resistance through adenosine pathway activation and represent a potential target for future studies.

## Main

Prostate cancer is the most prevalent male malignancy, with approximately 290,000 new cases diagnosed and 35,000 deaths per year in the USA^5^. Androgen deprivation therapy (ADT) initially elicits clinical responses, but most patients with advanced prostate cancer eventually progress to mCRPC and succumb to this disease^6^. There is therefore a clinical need to develop more effective treatment options. In recent years, ICIs have been approved for the treatment of multiple cancer types by disrupting checkpoint proteins, including cytotoxic lymphocyte antigen 4 (CTLA-4), PD-1 and PD-L1 (ref. ^7^). However, despite sporadic clinical responses largely restricted to rare molecular subtypes^8^, patients with mCRPC are typically refractory to these modalities^1,2^, underscoring the need for more therapeutic strategies that address the mechanisms of resistance in tumours^9,10^. However, these approaches have faced substantial problems, largely resulting from our limited understanding of the complex and intricate nature of mCRPC tumours.

The tumour microenvironment (TME), which is established by bidirectional interactions between tumour cells and components of their local environments, is a critical factor in promoting immunotherapeutic resistance across multiple malignancies^11,12^. In prostate cancer, numerous cellular components and soluble factors contribute to the establishment of an immunosuppressive niche^13,14^. Notably, there is substantial evidence that myeloid cells, particularly tumour-associated macrophages (TAMs) and myeloid-derived suppressor cells (MDSCs), mediate immunosuppression in prostate cancer by multiple mechanisms^3,4^. In particular, the abundance of these suppressive myeloid cells is significantly increased after ADT^15,16^, implying that they have a role as drivers of immunotherapy resistance in mCRPC. Although targeting myeloid cells has shown promise in enhancing the efficacy of ICIs in preclinical models^17,18^, translating these findings into clinical applications, through broad myeloid-targeted interventions such as the blockade of CSF1R^19,20^, has not demonstrated significant efficacy in either improving antitumour responses or providing substantial benefits to a wide subset of patients. This finding is attributed, in part, to the inherent heterogeneity of the targeted populations^10,13^. We therefore propose that a comprehensive understanding of specific immunosuppressive myeloid subsets that are highly enriched in the advanced stages of prostate cancer could result in more effective disruption of their molecular mechanisms, enhancing the efficacy of immunotherapy.

In recent years, multi-omics single-cell profiling technologies have revolutionized our understanding of the heterogeneity of the TME across multiple malignancies at the single-cell level^21–24^. These techniques have revealed previously unknown cell types and states within the prostate TME that mediate immunosuppression. For example, studies have identified cells such as fibroblasts that produce C-C motif chemokine ligand 2 (CCL2) and C-X-C motif chemokine ligand 12 (CXCL12)^25^, as well as endothelial cells and pericytes that enhance the dysregulation of angiogenesis^26^. Single-cell assessment has also been used to investigate the diverse population of tumour-infiltrating myeloid cells in either primary or metastatic prostate cancer^27–30^. This research has identified the molecular mechanisms of immunosuppression mediated by myeloid cells, such as the activation of a CCR6–CCL20 axis by inflammatory monocytes and M2 macrophages residing in bone metastases^28^. However, previous single-cell immune profiling of the prostate TME has predominantly focused on lymphocytes, particularly T cells, leaving a substantial gap in our understanding of the complexity of myeloid cells. Moreover, our knowledge of the evolution of the myeloid-mediated mechanisms underlying immunosuppression as prostate cancer progresses remains limited.

Here, we report that the myeloid-mediated mechanisms of immunotherapy resistance evolve as prostate cancer progresses. Through single-cell transcriptional profiling of patient biopsies, we identify a distinct macrophage subset characterized by elevated *SPP1* transcript levels (referred to as *SPP1*^hi^-TAMs), which becomes increasingly abundant with elevated immune inhibitory molecular programs as the disease advances. Notably, this specific macrophage population expresses reduced levels of *CSF1R* transcripts, indicating a potential link to the clinical ineffectiveness of CSF1R blockade in prostate cancer treatment. We reverse translate our findings to a syngeneic CRPC mouse model, in which we find an analogous macrophage subset through single-cell assessment. We demonstrate its role as a driver of immunotherapy resistance by computational analysis, functional assays and adoptive transfer experiments. We also find that *SPP1*^hi^-TAMs directly suppress T cells through the activation of the adenosine signalling pathway. Inhibiting this pathway significantly reduces tumour growth and sensitizes tumour cells to ICI therapies in both humans and mice. Consistent with the findings from the mouse model, inhibition of A2AR using ciforadenant in combination with PD-L1 blockade with atezolizumab can induce clinical responses in patients with mCRPC. Collectively, these studies demonstrate that the myeloid-mediated mechanisms that underlie immunotherapeutic resistance evolve over the course of prostate cancer progression. *SPP1*^hi^-TAMs have a key role in suppressing antitumour activity by activating adenosine signalling in prostate cancer, potentially serving as biomarkers to predict therapeutic efficacy.

## Single-cell RNA-seq of human prostate cancer

**Fig. 1:**
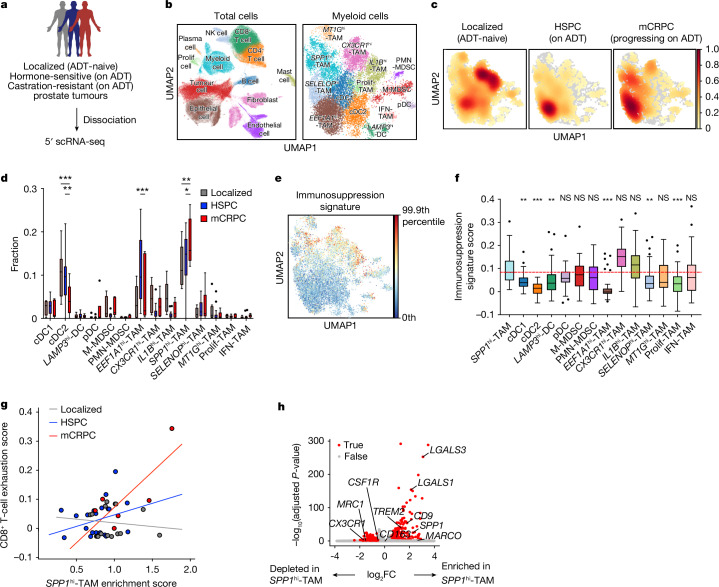
Single-cell assessment of biopsies from patients with prostate cancer reveals *SPP1*^hi^-TAMs with elevated immunosuppression programs prevalent in advanced disease stages. **a**, Schematic illustration of 5′ scRNA-seq (10x Genomics) on tumours from patients with either ADT-naive localized prostate cancer (*n* = 13), metastatic hormone-sensitive prostate cancer on ADT (HSPC; *n* = 24) or mCRPC progressing on ADT (*n* = 6). **b**, UMAP plots showing cell types (left) and distinct myeloid subsets (right) in human prostate cancer. Prolif, proliferative. **c**,**d**, Density (**c**) and bar plots (**d**) depicting the quantification of myeloid-subset frequencies across disease progression, with localized disease (grey; *n* = 13), HSPC (blue; *n* = 24) and mCRPC (red; *n* = 6). Significant changes were observed for cDC2 (*P* < 0.001 for mCRPC versus localized; *P* = 0.002 for mCRPC versus HSPC), *EEF1A1*^hi^-TAM (*P* < 0.001 for mCRPC versus HSPC) and *SPP1*^hi^-TAM (*P* = 0.002 for mCRPC versus localized; *P* = 0.04 for mCRPC versus HSPC). **e**,**f**, UMAP (**e**) and bar plots (**f**) showing immunosuppression gene signature scores among myeloid cells in human prostate cancer (*n* = 43 samples). In **d** and **f**, boxes represent the inter-quartile range (IQR), with bars indicating 25% − 1.5 × IQR and 75% + 1.5 × IQR. Outliers beyond 1.5 × IQR are included. The median score for *SPP1*^hi^-TAMs is indicated in red. **g**, Correlations between *SPP1*^hi^-TAM enrichment and CD8^+^ T cell exhaustion scores across disease stages. The lines represent the best-fit lines; each patient sample is indicated by a circle. HSPC, *P* = 0.17, *R* = 0.291; mCRPC, *P* = 0.07, *R* = 0.780; localized, *P* = 0.66, *R* = −0.134. **h**, Differentially expressed genes (adjusted *P* < 0.05, absolute log_2_ fold change (|log_2_FC|) > 0.5) in *SPP1*^hi^-TAMs compared with other myeloid cells highlighted in red. Statistical significance was determined by ordinary two-way analysis of variance (ANOVA) with Sidak correction (**d**); Kruskal–Wallis test with Dunn’s correction (**f**); simple linear regression analyses (**g**); and Wilcoxon test with Benjamini–Hochberg correction (**h**).*P < 0.05, **P < 0.01, ***P < 0.001; NS, not significant. The illustration in a was created using BioRender (https://biorender.com).

**Extended Data Fig. 1:**
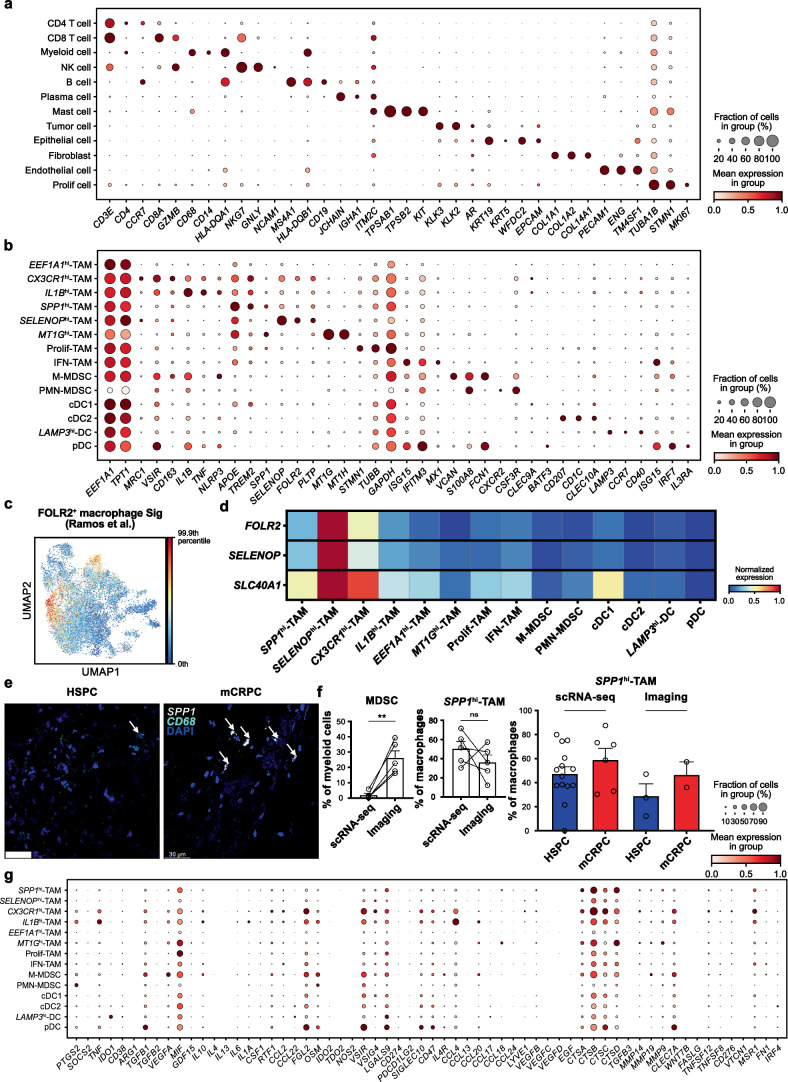
Single-cell analysis of prostate cancer patient biopsies identifies diverse cell types including multiple myeloid subsets. (a-b) Bubble plots showing the relative expression levels of signature genes for (a) the indicated major cell types and (b) myeloid subsets. (c-d) (c) UMAP plot showing the enrichment scores for published FOLR2^+^ macrophage signatures and (d) heatmap depicting the normalized expression levels of *FOLR2*, *SELENOP*, and *SLC40A1* in the indicated myeloid subsets. (e) Representative RNAscope images of HSPC and mCRPC patient biopsy samples. Immunostaining for *SPP1*^hi^-TAMs (*SPP1*; white, *CD68*; turquoise, and DAPI; blue) is presented. Scale bars, 30 μm. (f) Quantitative comparison of MDSC (*P* = 0.004) and *SPP1*^hi^-TAM (*P* = 0.34) frequency between scRNA-seq analysis versus tissue imaging. Bars show mean + SEM; symbols represent individual patients. (g) Bubble plot showing the relative expression levels of signature genes associated with immunosuppression. Statistical significance was determined by (f) two-sided paired Student’s t-tests; *P*-values: **P < 0.01. ns, not significant.

**Extended Data Fig. 2:**
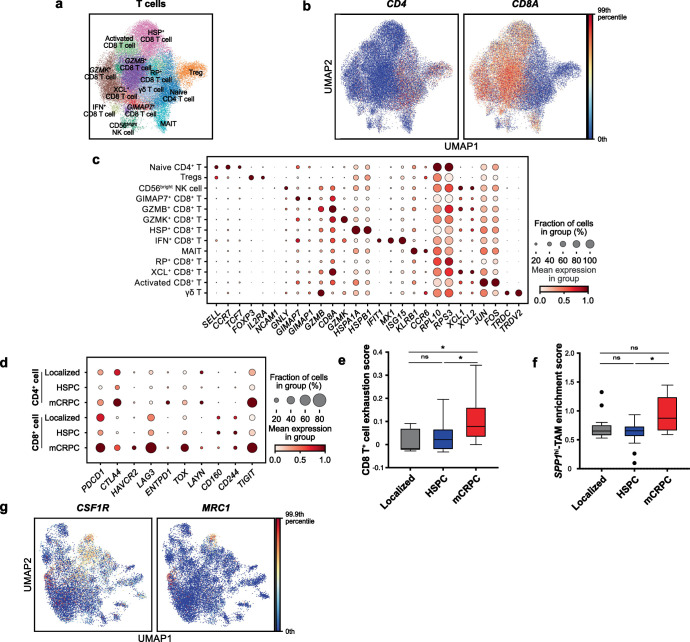
CD8^+^ T cells exhibit significant exhaustion in mCRPC, coinciding with a notable increase in the abundance of *SPP1*^hi^-TAMs. (a-b) UMAP plots showing (a) distinct T-cell subsets and (b) the relative expression levels of *CD4* and *CD8* transcripts across T cells in human prostate cancer. (c-d) Bubble plots depicting the relative expression levels of signature genes for (c) the indicated T-cell subsets and (d) exhaustion states in CD4^+^ or CD8^+^ T cells across different disease stages, as indicated. (e-f) Quantification of enrichment scores for (e) CD8^+^ T-cell exhaustion and (f) *SPP1*^hi^-TAMs across different disease stages, indicated by localized disease (n = 13), HSPC (n = 24), and mCRPC (n = 6) in gray, blue, and red, respectively. Boxes denote inter-quartile range (IQR), while bars denote 25% - 1.5 x IQR and 75% + 1.5 x IQR, with outliers exceeding 1.5 x the IQR beyond lower and upper quartiles. In (e), *P* = 0.96, 0.03, and 0.049 for localized vs. HSPC, localized vs. mCRPC, and HSPC vs. mCRPC, respectively. In (f), *P* = 0.51, 0.16, and 0.01 for localized vs. HSPC, localized vs. mCRPC, and HSPC vs. mCRPC, respectively. (g) UMAP plots showing the relative expression levels of *CSF1R* and *MRC1* transcripts across myeloid cells in human prostate cancer. Statistical significance was determined by (e, f) ordinary one-way ANOVA with the Sidak correction; *P*-values: *<0.05. ns, not significant.

To investigate the myeloid compartment and identify distinct immunosuppressive subsets during disease progression at the single-cell level, we used single-cell RNA (scRNA)-seq through a droplet-based 5′ 10x Genomics platform on tumour biopsies from patients with prostate cancer at various stages, including those with ADT-naive localized disease, metastatic hormone-sensitive prostate cancer (HSPC) on ADT, or mCRPC progressing on ADT (Fig. 1a). After rigorous quality control and data filtering, we obtained 147,174 single-cell transcriptomes. Using differentially expressed genes, we defined tumour cells and the major components of the TME, including immune cells and stromal cells (Fig. 1b and Extended Data Fig. 1a). Unsupervised clustering further identified 14 distinct subsets of tumour-infiltrating myeloid cells, including eight macrophage subsets, two MDSC subsets, three DC subsets and plasmacytoid DCs (pDCs) (Fig. 1b and Extended Data Fig. 1b). Our analysis revealed dynamic changes in the myeloid compartment as the disease progressed. For example, consistent with previous studies^15,16^, we observed enrichment of MDSCs and TAMs with elevated expression of *CX3CR1* and *CD163* (*CX3CR1*^hi^-TAMs; Extended Data Fig. 1b) in mCRPC compared with HSPC, although this trend was not statistically significant (Fig. 1c,d and Extended Data Fig. 1b). We also identified a distinct TAM subset that exhibited elevated enrichment scores for published FOLR2^+^ macrophage signatures^31^, including *SELENOP*, *FOLR2* and *SLC40A1* transcripts (referred to as *SELENOP* ^hi^-TAMs in this study), which is associated with CD8^+^ T cell infiltration and improved patient prognosis in human breast cancer^31^, although their abundance did not significantly change with disease progression (Fig. 1c,d and Extended Data Fig. 1b–d). As well as these populations, we found a significant increase in macrophages characterized by elevated *SPP1* transcript levels (*SPP1*^hi^-TAMs) during disease progression (Fig. 1c,d and Extended Data Fig. 1b). Their presence was further confirmed by tissue staining from patients with either HSPC or mCRPC (Extended Data Fig. 1e). Droplet-based scRNA-seq is recognized to have technical limitations in capturing fragile populations, such as neutrophils and MDSCs^32^. This was evident in our comparison of the scRNA-seq data with staining of matched patient tissues using anti-human CD11b and CD15 antibodies (Extended Data Fig. 1f). Nevertheless, we confirmed an increased abundance of *SPP1*^hi^-TAMs during disease progression (Extended Data Fig. 1e,f) through tissue staining of matched patient tissues, which is consistent with the scRNA-seq results. This result indicates that the prevalence of *SPP1*^hi^-TAMs in mCRPC reflects the cellular composition of the prostate TME. Given their increased prevalence, we hypothesized that this macrophage population could have a critical role in mediating immunotherapy resistance in mCRPC. Consistent with this, we found that these *SPP1*^hi^-TAMs exhibited elevated immunosuppression molecular programs relative to other myeloid subsets^21,33–35^ (Fig. 1e,f and Extended Data Fig. 1g). Furthermore, our analysis of the T cell compartment (Extended Data Fig. 2a–c) revealed that elevated *SPP1*^hi^-TAM gene signatures were significantly correlated with the degree of CD8^+^ T cell exhaustion^36^, which showed a marked increase as the disease advanced (Fig. 1g and Extended Data Fig. 2d–f). Notably, further transcriptional analysis revealed a significant decrease in *CSF1R* transcript levels in *SPP1*^hi^-TAMs relative to other myeloid cells (Fig. 1h and Extended Data Fig. 2g), indicating a mechanism that could contribute to the ineffectiveness of CSF1R blockade. Collectively, these results demonstrate that progression of prostate cancer leads to dynamic changes in the myeloid landscape within the TME, where *SPP1*^hi^-TAMs emerge as potential drivers of immunotherapeutic resistance.

## *Spp1*^hi^-TAMs in mouse prostate cancer

**Fig. 2:**
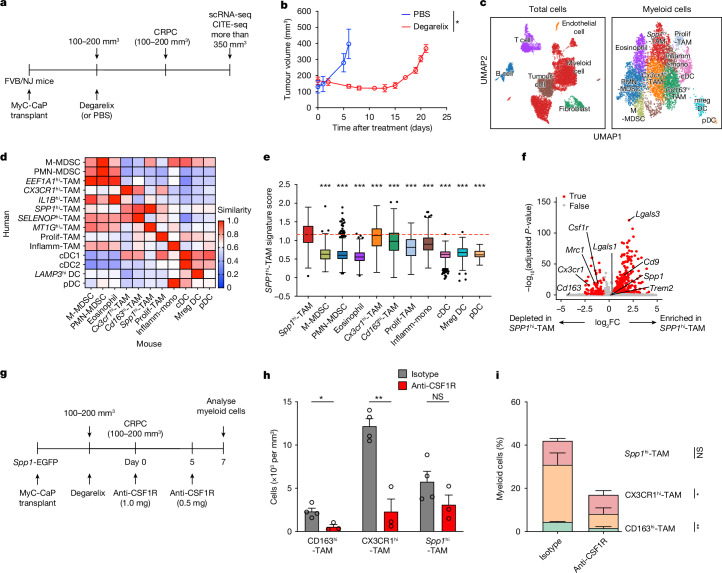
*Spp1*^hi^-TAMs in mouse prostate cancer are identified through scRNA-seq and demonstrate resistance to CSF1R blockade. **a**, Schematic of 5′ scRNA-seq (10x Genomics) and CITE-seq (cellular indexing of transcriptomes and epitopes by sequencing) on immune (CD45^+^) and non-immune (CD45^−^) cells from mouse prostate cancer (MyC-CaP), subcutaneously engrafted on mice treated with degarelix or PBS. **b**, Cumulative MyC-CaP growth in mice, comparing degarelix-treated (red; *n* = 3) and PBS-treated (blue; *n* = 3) groups (*P* = 0.046). Symbols show mean ± s.e.m. **c**, UMAP plots showing the main cell types (left) and distinct myeloid subsets (right) in mouse prostate cancer. Prolif, proliferative; Inflamm, inflammatory; mono, monocytes. **d**, Heatmap comparing myeloid subset similarity scores between human (rows) and mouse (columns) prostate cancer. **e**, *SPP1*^hi^-TAM signature scores across myeloid cells (*n* = 6,397 cells) in mouse prostate cancer (*P* < 0.001 for comparisons of *Spp1*^hi^-TAM versus each subset). Enrichment scores were calculated using gene signatures in the patient dataset shown in Fig. 1. The red dashed line shows the median score for *Spp1*^hi^-TAMs for comparison. Boxes denote IQR; bars show 25% − 1.5 × IQR and 75% + 1.5 × IQR, with outliers exceeding 1.5 × IQR. **f**, Plot of differentially expressed genes (adjusted *P*-value < 0.05, |log_2_FC| > 0.5) (red), indicating enrichment or depletion in *Spp1*^hi^-TAMs versus other macrophages and monocytes. **g**, Schematic of anti-CSF1R or isotype-matched control antibody dosing in *Spp1*-EGFP mice after CRPC development, assessing myeloid composition 2 days after treatment. **h**,**i**, Quantification of cell number (**h**) and frequency (**i**) for macrophage subsets in CRPC mice treated with anti-CSF1R (*n* = 3) or isotype-matched control (*n* = 4) antibodies. Bars show mean + s.e.m. from 3 independent experiments; symbols represent individual mice. Significant changes were observed in CD163^hi^-TAM and CX3CR1^hi^-TAM populations (*P* = 0.02, *P* = 0.002 (**h**); *P* = 0.003, *P* = 0.03 (**i**), but not in *Spp1*^hi^-TAMs (*P* = 0.18, *P* = 0.30). Statistical significance was determined by two-sided unpaired Student’s *t*-tests (**b**,**h**,**i**), Kruskal–Wallis test with Dunn’s correction (**e**) and Wilcoxon test with Benjamini–Hochberg correction (**f**); *P < 0.05, **P < 0.01, ***P < 0.001; NS, not significant. Source Data

**Fig. 3:**
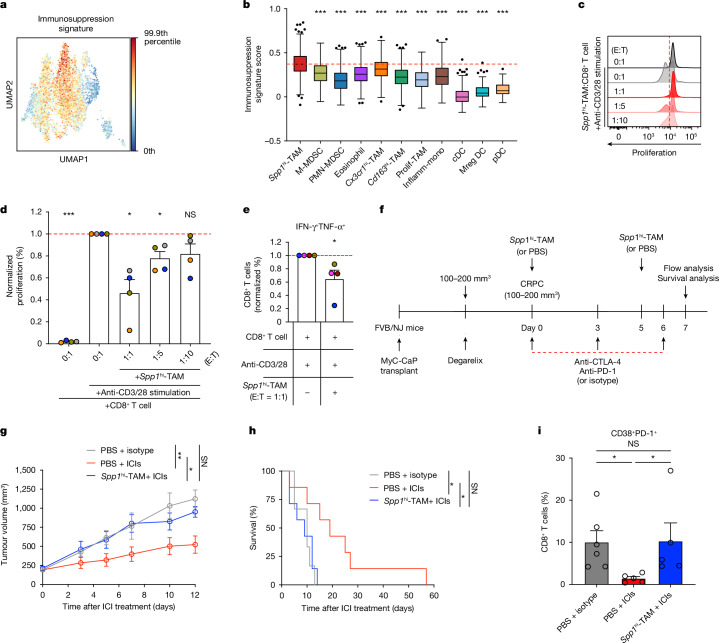
*Spp1*^hi^-TAMs have a critical role in promoting immunotherapeutic resistance by inducing exhaustion in CD8^+^ T cells in vivo. **a**,**b**, UMAP (**a**) and bar plots (**b**) showing immunosuppression scores among myeloid cells in mouse prostate cancer (*n* = 6,397; *P* < 0.001 for comparisons of *Spp1*^hi^-TAM and other subsets). Boxes represent IQR and bars indicate 25% − 1.5 × IQR and 75% + 1.5 × IQR, with outliers beyond 1.5 × IQR. The red dashed line shows the median score for *Spp1*^hi^-TAMs. **c**, Flow-cytometry plots showing reduced proliferation of activated splenic CD8^+^ T cells 3 days after co-culturing with *Spp1*^hi^-TAMs from CRPC. **d**,**e**, Quantification of proliferating (*P* = 0.02, *P* = 0.04 and *P* = 0.14 for effector:target (E:T) ratios of 1:1, 1:5 and 1:10, respectively (**d**) and polyfunctional (IFN-γ^+^TNF-α^+^; *P* = 0.01) CD8^+^ T cells with and without *Spp1*^hi^-TAMs at various ratios (**e**). Results are normalized to activated T cells alone; mean + s.e.m. from *n* = 4 experiments, with different colours for each and symbols for averages of 2–3 replicate wells. Red dashed lines indicate the normalized mean frequency of activated CD8^+^ T cells. **f**, Dosing schedule for ICIs (anti-CTLA-4 + anti-PD-1) or isotype-matched controls after adoptive transfer of *Spp1*^hi^-TAMs or PBS into CRPC. **g**, CRPC growth curves for ICI or isotype treatments after *Spp1*^hi^-TAM or PBS transfer from *n* = 3 experiments (*P* = 0.002, *P*  =  0.02 and *P*  =  0.59 for PBS+isotype versus PBS + ICIs, PBS + ICIs versus *Spp1*^hi^-TAM + ICIs and PBS + isotype versus *Spp1*^hi^-TAM + ICIs, respectively); PBS + isotype, *n* = 6; PBS + ICIs, *n* = 7; *Spp1*^hi^-TAM + ICIs, *n* = 7. Symbols represent mean ± s.e.m. **h**, Survival curves from the same experiment as **g** (*P* = 0.023, *P*  =  0.013 and *P*  =  0.755). **i**, Exhausted (CD38^+^PD-1^+^) CD8^+^ T cell frequencies in CRPC after *Spp1*^hi^-TAMs or PBS transfer with or without ICIs, assessed 1 day after the final injection (*P* = 0.02, *P*  =  0.02, *P*   > 0.99). Bars show mean + s.e.m. from *n* = 3 experiments; symbols represent individual mice. Statistical significance was determined by Kruskal–Wallis tests with Dunn’s correction (**b**,**i**), two-sided one-sample *t*-tests (**d**,**e**), ordinary one-way ANOVA with Sidak correction (**g**) and log-rank tests (**h**); *P < 0.05, **P < 0.01, ***P < 0.001; NS, not significant. Source Data

**Extended Data Fig. 3:**
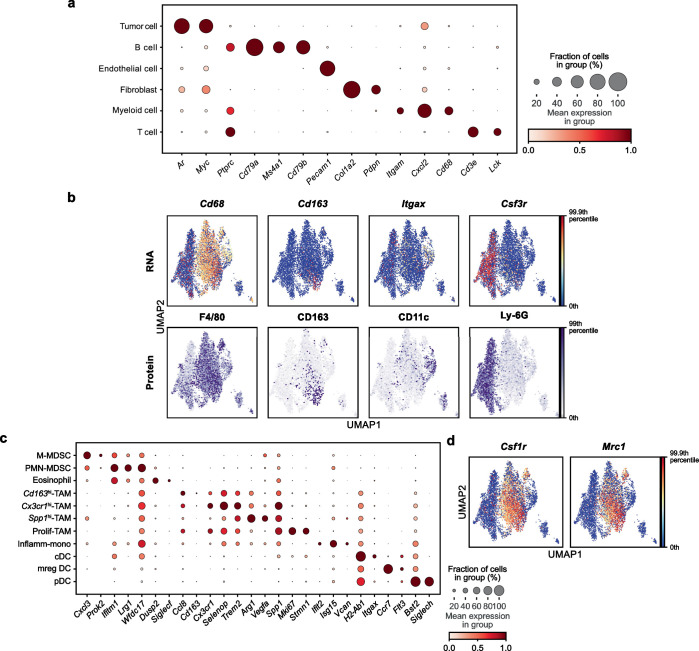
Single-cell analysis of mouse prostate cancer reveals diverse cell types including multiple myeloid subsets. (a) Bubble plot showing the relative expression levels of signature genes for the indicated major cell types in mouse prostate cancer. (b) UMAP plots showing the relative expression levels of *Cd68, Cd163, Itgax,* and *Csf3r* transcripts (top) and the protein levels of F4/80, CD163, CD11c, and Ly-6G (bottom) across myeloid cells. (c) Bubble plot depicting the relative expression levels of signature genes for the indicated myeloid subsets. (d) UMAP plots showing the relative expression levels of *Csf1r* and *Mrc1* transcripts across myeloid cells.

**Extended Data Fig. 4:**
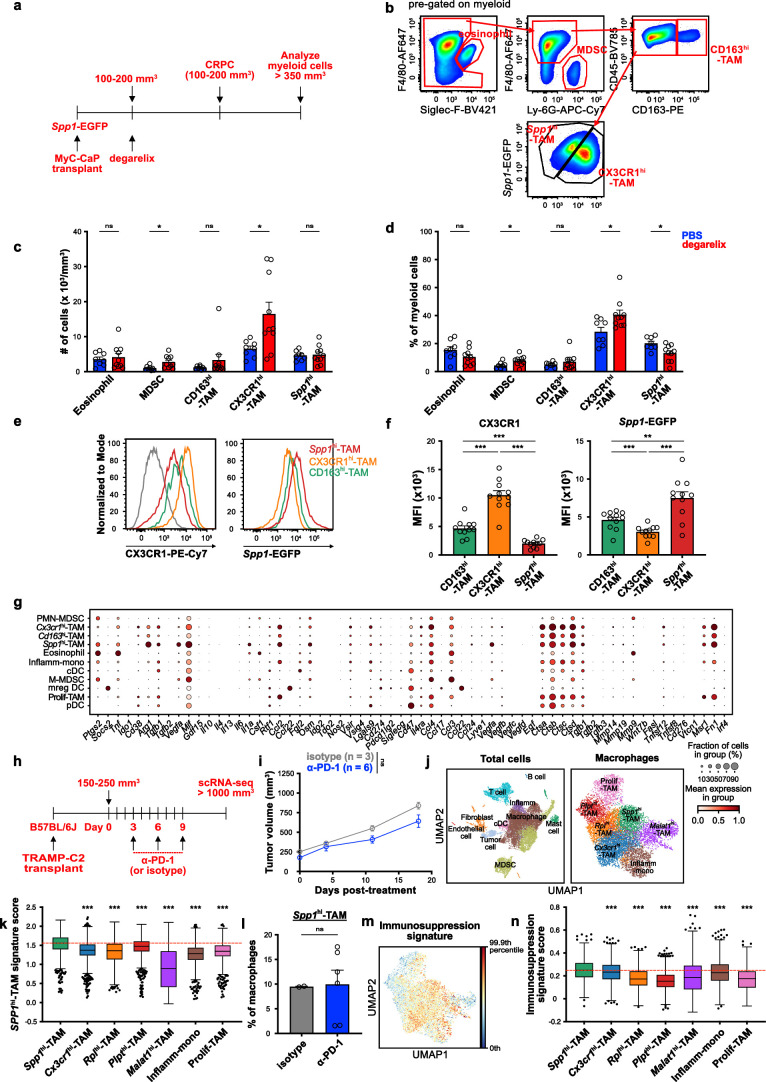
Flow cytometry analysis of the myeloid composition in MyC-CaP-derived HSPC and CRPC developed in *Spp1*-EGFP mice, and single-cell assessment of the myeloid landscape in TRAMP-C2. (a) Experimental schematic depicting the evaluation of the myeloid compartment in CRPC developed in *Spp1*-EGFP mice following degarelix treatment. (b) Representative sequential gating schemes for evaluation of the indicated myeloid subsets in mice bearing CRPC. The initial plot is pre-gated on live, singlet, myeloid (CD11b^+^) cells. Sequential gating is indicated by arrows. (c-d) Quantification of the (c) number and (d) frequency of the indicated myeloid subsets from MyC-CaP engrafted into mice treated with degarelix (CRPC; red; n = 10) or PBS control (HSPC; blue; n = 8). In (c), *P* = 0.70, 0.02, 0.36, 0.02, and 0.84 for eosinophil, MDSC, CD163^hi^-TAM, CX3CR1^hi^-TAM, and *Spp1*^hi^-TAM, respectively. In (d), *P* = 0.11, 0.02, 0.70, 0.049, and 0.01 for eosinophil, MDSC, CD163^hi^-TAM, CX3CR1^hi^-TAM, and *Spp1*^hi^-TAM, respectively. Bars represent the mean + SEM from 4 independent experiments; symbols represent individual mice from each experiment. (e-f) (e) Representative flow cytometry plots and (f) quantification of cell surface CX3CR1 (left) or intracellular *Spp1* (right) expression levels of the indicated macrophage subsets from CRPC (n = 11). Isotype control stains are in gray. In (f), *P* < 0.001 for all comparisons, except for *Spp1*-EGFP MFI between CD163^hi^-TAM and *Spp1*^hi^-TAM, where *P* = 0.002. Bars represent the mean + SEM from the same experiments as in (c, d); symbols represent individual mice from each experiment. (g) Bubble plot showing the relative expression levels of signature genes associated with immunosuppression. (h) Experimental schematic depicting 10x Genomics 5’ scRNA-seq on immune (CD45^+^) and non-immune (CD45^-^) cells isolated from mice subcutaneously engrafted with mouse prostate cancer, TRAMP-C2, followed by treatment with anti-PD-1 or isotype antibodies. (i) Graph shows the cumulative growth of TRAMP-C2 engrafted into healthy wild-type mice, where blue and gray represent groups treated with anti-PD-1 antibody (n = 6) and isotype antibody (n = 3), respectively. Symbols represent mean +/- SEM from 2 independent experiments. (j) UMAP plots showing major cell types (left) and distinct myeloid subsets (right) in TRAMP-C2. (k-l) Quantification of (k) signature scores for *SPP*1^hi^-TAMs across macrophages and monocytes (n = 9,460 cells) and (l) the frequency of *Spp*1^hi^-TAMs in TRAMP-C2 engrafted in mice treated with either anti-PD-1 or isotype control antibodies. In (k), *P* < 0.001 for all comparisons between *Spp1*^hi^-TAM vs. each indicated subset. Boxes denote IQR, while bars denote 25% - 1.5 x IQR and 75% + 1.5 x IQR, with outliers exceeding 1.5 x the IQR beyond lower and upper quartiles. In (l), bars show mean + SEM; symbols represent individual mice (*P* = 0.93). The red line indicates the median score of *Spp*1^hi^-TAMs. (m-n) (m) UMAP and (n) bar plots showing the quantification of immunosuppression gene signature scores among macrophages and monocytes in TRAMP-C2 (n = 9,460 cells). In (n), *P* < 0.001 for all comparisons between *Spp1*^hi^-TAM vs. each indicated subset. Boxes denote IQR, while bars denote 25% - 1.5 x IQR and 75% + 1.5 x IQR, with outliers exceeding 1.5 x the IQR beyond lower and upper quartiles. The red line indicates the median score of *Spp*1^hi^-TAMs. Statistical significance was determined by (c, d, l) two-sided unpaired Student’s t-tests, (f) repeated measures one-way ANOVA with the Sidak correction, (i) a log-rank test, and (k, n) Kruskal-Wallis tests with the Dunn’s correction; *P*-values: *<0.05, **P < 0.01, ***<0.001. ns, not significant.

MyC-CaP is a prostate cancer cell line that is dependent on androgens and originates from a male mouse with prostate cancer^37^. To delve deeper into our findings from patients, we performed droplet-based 5′ scRNA-seq (10x Genomics) with characterization of myeloid cell-surface antigens, including F4/80, CD11c, CD163 and Ly-6G, on a 1:1 mixture of fluorescence-activated cell sorting (FACS)-isolated immune (CD45^+^) and non-immune (CD45^−^) cells from mice subcutaneously engrafted with MyC-CaP, followed by treatment with either degarelix acetate (a gonadotropin-releasing hormone antagonist) or phosphate-buffered saline (PBS) (Fig. 2a). Consistent with previous studies^27,38^, tumour burden significantly regressed after degarelix treatment and then progressed as CRPC (Fig. 2b). By using scRNA-seq, we identified 6 main cell types in the TME, along with 11 distinct myeloid subsets (Fig. 2c and Extended Data Fig. 3a–c). Comparative analysis of transcriptomes between humans and mice enabled us to identify a mouse macrophage subset (*Spp1*^hi^-TAMs) that is analogous to human *SPP1*^hi^-TAMs) (Fig. 2d,e), characterized by increased expression of *Spp1*, *Cd9* and *Lgals3* transcripts and reduced expression of *Csf1r, Mrc1*, *Cx3cr1* and *Cd163* (Fig. 2f and Extended Data Fig. 3d). To validate these transcriptional findings at the protein level, we established CRPC in *Spp1*-EGFP mice, in which EGFP is expressed under the control of the *Spp1* promotor (Extended Data Fig. 4a). By using a multi-parameter flow-cytometry panel (Extended Data Fig. 4b), we confirmed the presence of multiple myeloid subsets identified through scRNA-seq and observed dynamic changes in the myeloid composition (Extended Data Fig. 4c–f). We observed that the cellularity of *Spp1*^hi^-TAMs remained largely consistent between HSPC and CRPC, although their frequency decreased during disease progression, mainly because of significant infiltration by *Cx3cr1*^hi^-TAMs (Extended Data Fig. 4c,d), as reported previously^15^. To determine whether *Spp1*^hi^-TAMs are resistant to CSF1R blockade, we administered an anti-CSF1R antibody to mice bearing CRPC (Fig. 2g). In line with our transcriptional findings, macrophages with high expression of *Csf1r* transcripts (Extended Data Fig. 3d), including *CD163*^hi^-TAMs and *CX3CR1*^hi^-TAMs, were significantly ablated, but *Spp1*^hi^-TAMs remained largely unaffected (Fig. 2h,i). This supports a potential role of *SPP1*^hi^-TAMs in contributing to the therapeutic resistance of CSF1R blockade. Consistent with data from humans, *Spp1*^hi^-TAMs in mouse prostate cancer exhibited significantly elevated immunosuppressive gene signatures compared with other myeloid cells (Fig. 3a,b and Extended Data Fig. 4g), supporting their role as drivers of immunotherapy resistance. We found analogous results with the TRAMP-C2 model^39^, a syngeneic prostate cancer cell line that, unlike MyC-CaP, is not *Myc*-driven, treated with either anti-PD-1 or isotype-matched control antibodies (Extended Data Fig. 4h). Consistent with previous studies^40^, anti-PD-1 antibody treatment alone showed minimal efficacy in this model (Extended Data Fig. 4i). Single-cell analysis revealed the presence of *Spp1*^hi^-TAMs with elevated immunosuppressive molecular programs relative to other TAM subsets, in line with the MyC-CaP model (Extended Data Fig. 4j–n). Taken together, through single-cell assessment, our data enabled us to identify analogous *Spp1*^hi^-TAMs with elevated immunosuppressive gene signatures across multiple mouse models of prostate cancer, and we subsequently demonstrated their resistance to CSF1R inhibition.

## *Spp1*^hi^-TAMs drive immunotherapy resistance

**Extended Data Fig. 5:**
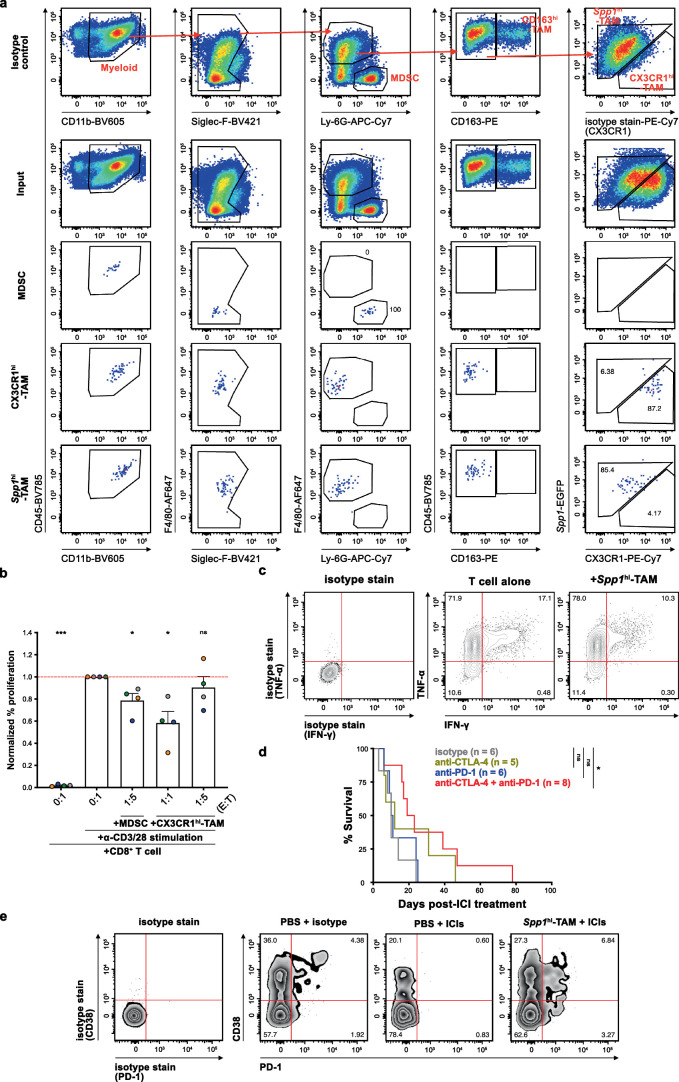
Functional assays demonstrating the immune suppressive activity of myeloid cells in CRPC, and a significant increase in exhausted CD8^+^ T cells following the transfer of *Spp1*^hi^-TAMs combined with CTLA-4 and PD-1 blockade in vivo. (a) Representative flow cytometry plots showing the purity of each myeloid subset after FACS sorting. Myeloid subsets were gated based on the strategy shown at the top, with sequential gating strategies indicated by arrows. (b) Quantification of the frequency of proliferating CD8^+^ T cells 3 days after co-culture with either MDSC (*P* = 0.04) or CX3CR1^hi^-TAMs (*P* = 0.03 and 0.14 for E:T = 1:1 and 1:5, respectively) FACS-isolated from CRPC at the indicated ratios. Results were normalized to cultures with activated T cells alone. Bars show the mean + SEM from 4 independent experiments, each indicated by a distinct color; symbols represent averages of 2-3 technical replicate wells. Red lines indicate the normalized mean frequency of activated CD8^+^ T cells cultured alone. (c) Representative flow cytometry plots showing a decrease in the frequency of polyfunctional (IFN-γ^+^TNF-α^+^) CD8^+^ T cells 3 days after co-culture with *Spp1*^hi^-TAMs at a 1:1 ratio from the same experiments as in Fig. 3e. (d) Graph depicting the cumulative growth of CRPC following treatment with anti-CTLA-4 +/- anti-PD-1 antibodies (*P* = 0.33, 0.49, and 0.04 for isotype vs. anti-CTLA-4, anti-PD-1, and anti-CTLA-4 + anti-PD-1, respectively). (e) Representative flow cytometry plots showing an increased frequency of exhausted (CD38^+^PD-1^+^) CD8^+^ T cells following the transfer of *Spp1*^hi^-TAMs in combination with anti-CTLA-4 and anti-PD-1 antibodies, from the same experiments as in Fig. 3i. Statistical significance was determined by (b) one sample t-tests, and (d) log-rank tests; *P*-values: *<0.05, ***<0.001. ns, not significant.

To assess the ability of myeloid cells to functionally suppress T cell activity, multiple myeloid subsets, including MDSCs, *CX3CR1*^hi^-TAMs and *Spp1*^hi^-TAMs, were isolated by FACS from CRPC developed in *Spp1*-EGFP mice (Extended Data Fig. 5a) and co-cultured with splenic CD8^+^ T cells in the presence of anti-CD3/CD28 stimulation. As previously reported^18,41^, MDSCs and *CX3CR1*^hi^-TAMs effectively suppressed the proliferation of T cells in vitro, serving as controls (Extended Data Fig. 5b). Notably, we found that *Spp1*^hi^-TAMs significantly inhibited T cell proliferation in a density-dependent manner (Fig. 3c,d). Furthermore, the presence of *Spp1*^hi^-TAMs resulted in a marked decrease in the frequency of polyfunctional (IFN-γ^+^TNF-α^+^) CD8^+^ T cells (Fig. 3e and Extended Data Fig. 5c), indicating that their immunosuppressive activity can dampen T cell effector function. Next, we tested whether *Spp1*^hi^-TAMs can promote resistance to ICIs in vivo. We first confirmed that a combination of anti-CTLA-4 and anti-PD-1 antibodies results in a more significant decrease in the growth of CRPC than either given alone (Extended Data Fig. 5d), which is consistent with previous studies^27,38^. We reasoned that if *Spp1*^hi^-TAMs could mediate immunotherapy resistance, they would decrease the effectiveness of the dual treatment. To test this possibility, we adoptively transferred FACS-purified *Spp1*^hi^-TAMs into CRPC in the presence of the combination treatment, minimizing potential issues with their trafficking to the TME (Fig. 3f). Strikingly, intratumorally transferred *Spp1*^hi^-TAMs resulted in significantly diminished efficacy of the dual treatment and reduced overall survival (Fig. 3g,h). Transferring *Spp1*^hi^-TAMs significantly increased the frequency of exhausted (CD38^+^PD-1^+^) CD8^+^ T cells within ICI-treated tumours compared with control ICI-treated tumours. The levels of these exhausted T cells were similar to those observed in PBS-treated tumours (Fig. 3i and Extended Data Fig. 5e), highlighting the suppressive activity of *Spp*1^hi^-TAMs in vivo. Taken together, these results indicate that *Spp1*^hi^-TAMs have a critical role in driving immunotherapeutic resistance in CRPC.

## *SPP1*^hi^-TAMs drive suppression through adenosine

**Fig. 4:**
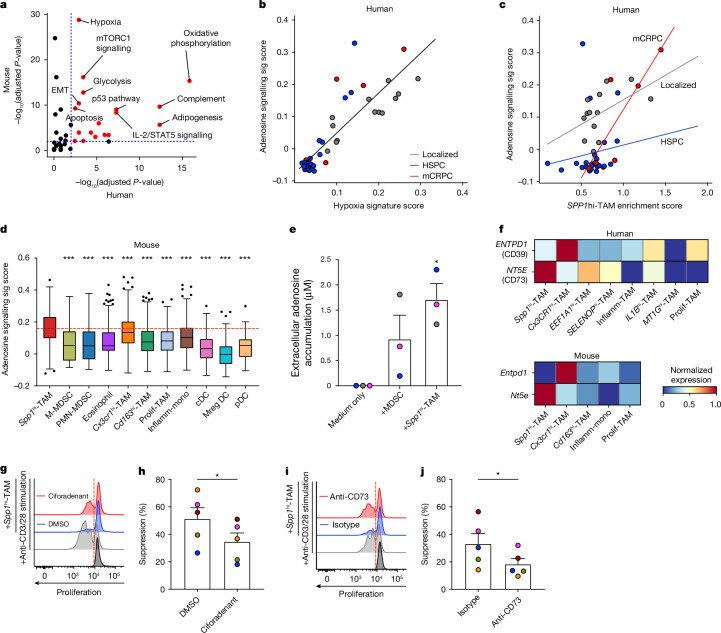
*SPP1*^hi^-TAMs are hypoxic and mediate immunosuppression through adenosine signalling. **a**, Enriched term clusters using differentially expressed genes (adjusted *P*-value < 0.05, |log_2_FC| > 0.5) in *SPP1*^hi^-TAMs versus other myeloid cells in humans and mice, using Enrichr with MSigDB Hallmark 2020 gene sets (blue dashed line at adjusted *P* = 0.05). **b**,**c**, Correlations between enrichment scores for hypoxia (*P* < 0.001, *R* = 0.858) (**b**) or *SPP1*^hi^-TAMs (**c**) and the adenosine signalling signature (sig) across patient samples with localized disease (grey, *P* = 0.08, *R* = 0.502), HSPC (blue, *P* = 0.54, *R* = 0.309) and mCRPC (red, P = 0.04, *R* = 0.839). Best-fit lines are shown, with symbols representing individual samples. **d**, Adenosine signalling signature scores in mouse prostate cancer myeloid cells (*n* = 6,397; *P* < 0.001 for *Spp1*^hi^-TAMs versus other subsets). Boxes denote IQR; bars indicate 25% − 1.5 × IQR and 75% + 1.5 × IQR, with outliers exceeding 1.5 × IQR. The red dashed line shows the median *Spp1*^hi^-TAM score. **e**, Extracellular adenosine accumulation by MDSCs or *Spp1*^hi^-TAMs after 1 day of culture, normalized to the background adenosine levels from medium without cells (*P* = 0.01). Bars show mean + s.e.m. from *n* = 3 experiments, with different colours for each and symbols for averages of 2 replicate wells. **f**, Heatmaps of normalized *ENTPD1* and *NT5E* expression in TAMs and monocytes from human (top) and mouse (bottom) prostate cancers. **g**,**h**, Flow cytometry (**g**) and bar plots (**h**) showing increased CD8^+^ T cell proliferation with *Spp1*^hi^-TAMs and ciforadenant (an A2AR inhibitor; 10 μM) versus DMSO (*P* = 0.04). **i**,**j**, Flow cytometry (**i**) and bar plots (**j**) showing increased CD8^+^ T cell proliferation with *Spp1*^hi^-TAMs and anti-CD73 antibody (10 μg ml^−1^) versus isotype-matched control antibody (*P* = 0.04). In **g**–**j**, bars show mean + s.e.m. from *n* = 5 independent experiments, each indicated by a different colour; symbols represent averages of 2–3 technical replicate wells. Statistical significance was determined by (Fisher’s exact and hypergeometric tests with Benjamini–Hochberg correction (**a**), simple linear regression analyses (**b**,**c**), a Kruskal–Wallis test with Dunn’s correction (**d**), two-sided one-sample *t*-tests (**e**) and two-sided paired Student’s *t*-tests (**h**,**j**); *P < 0.05, ***P < 0.001; NS, not significant. Source Data

**Extended Data Fig. 6:**
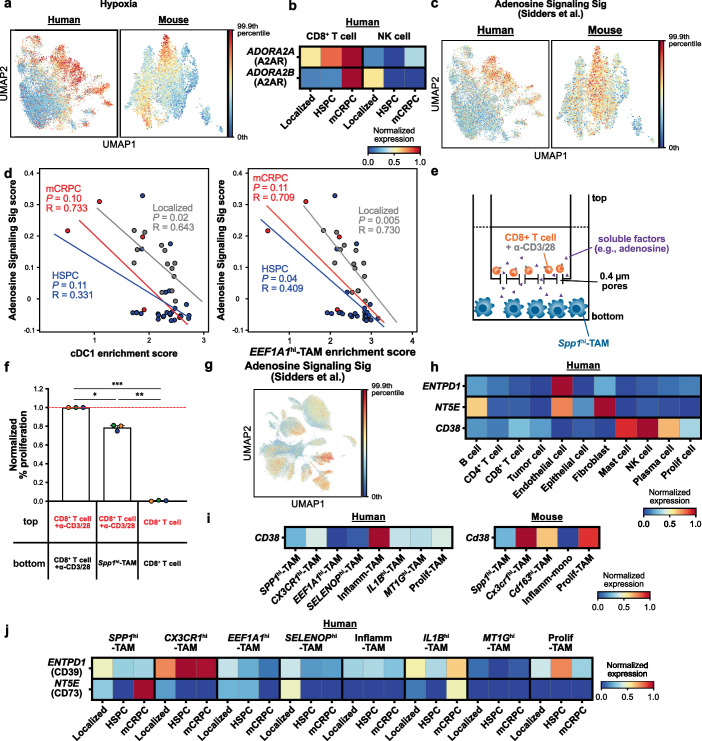
Evaluation of gene signatures related to adenosine signaling pathways across multiple cell types, including *SPP1*^hi^-TAMs, in human and mouse prostate cancers, and functional validation of the role of soluble factors in *SPP1*^hi^-TAM-mediated immunosuppression. (a) UMAP plots showing enrichment scores for the “Hypoxia” gene signatures across myeloid cells in both human and mouse prostate cancers. (b) Heatmaps depicting the normalized expression levels of *ADORA2A* and *ADORA2B* transcripts in the indicated CD8^+^ T cells and NK cells across different disease stages in patients. (c) UMAP plots showing enrichment scores for the “Adenosine Signaling Sig” gene signatures across myeloid cells in both human and mouse prostate cancers. (d) Plots depict the correlations between enrichment scores for the gene signatures “cDC1” (left) or “*EEF1A1*^hi^-TAMs” (right) and enrichment scores for “Adenosine Signaling Sig” across different disease stages in patient samples. Localized disease, HSPC, and mCRPC are in gray, blue, and red, respectively. The best-fit lines are displayed, and individual patient samples are represented by circles. (e) Schematic illustration of transwell assays in which CD8^+^ T cells, with or without α-CD3/28 stimulation, are cultured in the presence or absence of FACS-isolated *Spp1*^hi^-TAMs in opposite chambers to determine whether *Spp1*^hi^-TAMs suppress T-cell proliferation via soluble factors. (f) Quantification of T-cell proliferation in the red-indicated chamber 3 days after culture initiation (*P* = 0.02, 0.002, and <0.001 for comparisons between the left vs. middle, middle vs. right, and left vs. right, respectively, as shown in the schematic in (e)). Results were normalized to the proliferation of activated T cells cultured alone in the top chamber of the inserts within each experiment. Bars show the mean + SEM from 3 independent experiments, each indicated by a distinct color; symbols represent averages of 2-3 technical replicate wells. Red lines indicate the normalized mean of activated CD8^+^ T cells cultured alone. (g) UMAP plot showing enrichment scores for the “Adenosine Signaling Sig” gene signatures across major cell types in patients. (h) Heatmap depicting the normalized expression levels of *ENTPD1*, *NT5E*, and *CD38* transcripts in the indicated major cell types in patients. (i) Heatmaps depicting the normalized expression levels of *CD38* transcripts in the indicated myeloid subsets both in humans and mice. (j) Heatmaps depicting the normalized expression levels of *ENTPD1* and *NT5E* transcripts in the indicated tumor-associated macrophages across different disease stages in patients. Statistical significance was determined by (d) simple linear regression analyses, and (f) a repeated measures one-way ANOVA with the Sidak correction; *P*-values: *<0.05, **P < 0.01, ***<0.001. ns, not significant.

**Extended Data Fig. 7:**
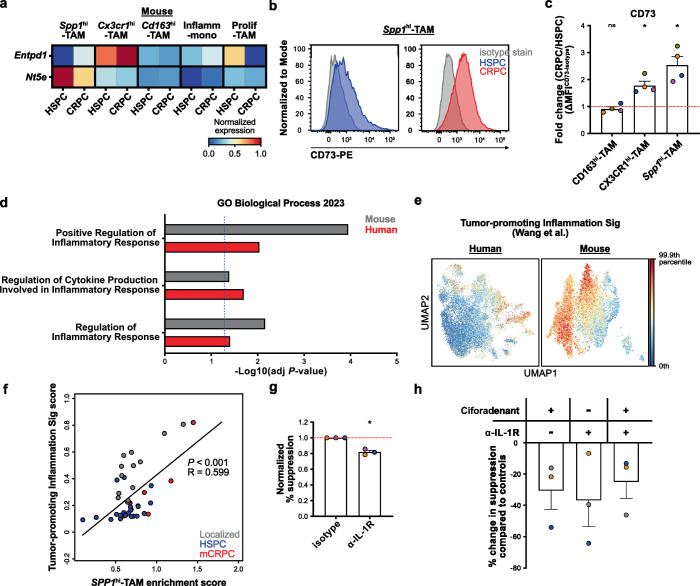
Assessment of CD39 and CD73 levels in *Spp1*^hi^-TAMs in mouse prostate cancer, and investigation of the role of the IL-1R pathway in immunosuppression mediated by *SPP1*^hi^-TAMs both in humans and mice. (a) Heatmap depicting the normalized expression levels of *Entpd1* and *Nt5e* transcripts in the indicated tumor-associated macrophages and monocytes across different disease stages in mice. (b-c) (b) Representative flow cytometry plots and (c) quantification of fold changes in the levels of cell surface CD73 expressed on *Spp1*^hi^-TAMs from CRPC relative to HSPC. HSPC, CRPC, and isotype control stains are shaded in blue, red, and gray, respectively in (b). In (c), *P* = 0.35, 0.01, and 0.01 for CD163^hi^-TAM, CX3CR1^hi^-TAM, and *Spp1*^hi^-TAM, respectively. Bars represent the mean + SEM from 4 independent experiments, each indicated by a distinct color; symbols represent individual mice from each experiment. The red line indicates a fold change of 1. (d) Pathways associated with inflammation, significantly enriched in *SPP1*^hi^-TAMs compared to other myeloid cells in both human (red) and mouse (gray) prostate cancers, were identified using the Enrichr bioinformatics tool with GO Biological Process 2023 gene sets. (e) UMAP plots showing enrichment scores for the “Tumor-promoting Inflammation” gene signatures across myeloid cells in both human and mouse prostate cancers. (f) Plot depicting the correlation between enrichment scores for the gene signatures “*SPP1*^hi^-TAMs” and enrichment scores for “Tumor-promoting Inflammation Sig” across different disease stages in patient samples. Localized disease, HSPC, and mCRPC are in gray, blue, and red, respectively. The best-fit line is displayed, and individual patient samples are represented by circles. (g) Bar plots showing decreased suppression of activated splenic CD8^+^ T cells when co-cultured with *Spp1*^hi^-TAMs in the presence of an anti-IL-1R antibody (10 μg/ml) compared to isotype-treated cultures (*P* = 0.01). Bars show mean + SEM from 3 independent experiments, each indicated by a distinct color; symbols represent averages of 2-3 technical replicate wells. (h) Bar plots showing the percentage change in suppression of activated splenic CD8^+^ T cells mediated by *Spp1*^hi^-TAMs in the presence of either ciforadenant (a A2AR inhibitor; 10 μM), anti-IL-1R antibody, or a combination of both. Bars show mean - SEM from 3 independent experiments, each indicated by a distinct color; symbols represent averages of 2-3 technical replicate wells. Statistical significance was determined by (c, g) two-sided one sample t-tests, (d) Fisher’s exact tests or the hypergeometric tests with the Benjamini-Hochberg correction, and (f) simple linear regression analysis; *P*-values: *<0.05. ns, not significant.

To explore the mechanisms by which *SPP1*^hi^-TAMs promote immunotherapeutic resistance, we further analysed our scRNA-seq datasets of human and mouse prostate cancers. Pathway analysis showed that hypoxia was among the top pathways activated preferentially in *SPP1*^hi^-TAMs in patients and mice (Fig. 4a and Extended Data Fig. 6a), which is consistent with previous studies that found that *SPP1* is upregulated in macrophages in the hypoxic TME^42^. Hypoxia is known to promote the accumulation of extracellular adenosine in tumours through the upregulation of CD39 and CD73, which are ectonucleotidases that convert ATP to ADP and AMP, and AMP to adenosine, respectively^43^. Alternatively, this process can also involve ectoenzymes such as CD38 and CD203a, which generate AMP by degrading NAD^+^ and ADPR^43^. Adenosine is an established mediator of immunosuppression in tumours^44^; its binding to adenosine receptors, particularly A2ARs and A2B receptors (A2BRs), which have higher and lower affinities, respectively, initiates downstream immunosuppressive signalling by the accumulation of intracellular cAMP, leading to the suppression of the antitumour activity of T cells and natural killer (NK) cells^45–47^. Notably, we observed elevated levels of *ADORA2A* transcripts, which encode A2ARs, during disease progression in both CD8^+^ T cells and NK cells, whereas *ADORA2B* transcript levels were increased only in CD8^+^ T cells (Extended Data Fig. 6b). Using the published specific gene signature associated with adenosine signalling^48^, which has been shown to strongly correlate with the extracellular adenosine concentration in the TME, we confirmed a strong correlation between enrichment scores for hypoxia and the adenosine signalling signature in our patient dataset (Fig. 4b), concordant with there being a link between hypoxia and adenosine accumulation. Notably, the expression of the genes associated with the adenosine signalling signature increasingly correlates with *SPP1*^hi^-TAM enrichment scores as the disease progresses (Fig. 4c and Extended Data Fig. 6c), but this trend is not evident in other myeloid populations, including *EEF1A1*^hi^-TAMs (Extended Data Fig. 6d). Consistent with human results, the adenosine signalling signature is elevated in *Spp1*^hi^-TAMs relative to other myeloid subsets in mice (Fig. 4d, Extended Data Fig. 6c). When stimulated splenic CD8^+^ T cells were cultured in transwell plates with *Spp1*^hi^-TAMs isolated by FACS, such that the two cell types were separated by micropores, allowing only soluble factors to pass through (Extended Data Fig. 6e), we observed that T cell proliferation was suppressed (Extended Data Fig. 6f). This finding indicates that the accumulation of soluble factors, including adenosine, may contribute to immunotherapeutic resistance mediated by *SPP1*^hi^-TAMs. Subsequent in vitro assays confirmed that *Spp1*^hi^-TAMs did indeed release extracellular adenosine (Fig. 4e). To further investigate the role of adenosine in immunosuppression mediated by *SPP1*^hi^-TAMs, we assessed the expression levels of *CD38*, *ENTPD1* and *NT5E*, which encode CD38, CD39 and CD73, respectively, across multiple cell populations with the emphasis on myeloid cells. Consistent with previous studies^43,49^, various cell types, including B cells and endothelial cells, exhibit an elevated adenosine signalling signature, with increased expression of *NT5E* and/or *ENTPD1* (Extended Data Fig. 6g, h). Notably, our analysis revealed elevated transcript levels of *NT5E*, but not *ENTPD1* or *CD38*, in *SPP1*^hi^-TAMs compared with other myeloid subsets in both humans and mice (Fig. 4f and Extended Data Fig. 6i). In particular, *NT5E* levels in *SPP1*^hi^-TAMs are significantly higher in mCRPC than in earlier stages (Extended Data Fig. 6j). This trend is similarly observed in CD73 protein levels in mice, although the transcript levels exhibited a diminished, but non-significant, change (Extended Data Fig. 7a–c). To test directly whether *SPP1*^hi^-TAMs suppress T cell activity through adenosine, splenic CD8^+^ T cells activated with anti-CD3/CD28 stimulation were co-cultured with *Spp1*^hi^-TAMs in the presence of ciforadenant, a small-molecule inhibitor of A2ARs (Fig. 4g,h), as well as a blocking antibody against CD73 (Fig. 4i,j). In both cases, blocking either the receptor or the ectoenzyme resulted in a significant reduction in suppression of T cells mediated by *Spp1*^hi^-TAMs, indicating that adenosine is closely involved in the immunosuppressive activity of *Spp1*^hi^-TAMs. However, inhibition of the adenosine pathway was not sufficient to fully restore T cell proliferation in culture, indicating a role for further mechanisms by which *Spp1*^hi^-TAMs can drive immunotherapeutic resistance. To investigate such suppressive mechanisms, we carried out further pathway analysis and identified multiple pathways enriched in *SPP1*^hi^-TAMs associated with inflammatory responses in both humans and mice (Extended Data Fig. 7d). These findings were supported by the elevated scores of published gene signatures for myeloid cells expressing proinflammatory soluble factors, such as IL-1β (tumour-promoting inflammation signature)^50^, in *SPP1*^hi^-TAMs across both species, and there was a significant correlation between *SPP1*^hi^-TAM abundance and tumour-promoting inflammation signature enrichment in patients (Extended Data Fig. 7e,f). Notably, blockade of IL-1R significantly diminished *Spp1*^hi^-TAM-mediated T cell suppression in culture (Extended Data Fig. 7g), indicating that IL-1R signalling also has an important role in driving immunotherapy resistance by these macrophages. There was no significant synergistic effect observed with the combined blockade of A2AR and IL-1R in vitro (Extended Data Fig. 7h). Collectively, these findings indicate that *SPP1*^hi^-TAMs dampen T cell activity, at least in part through extracellular adenosine.

## A2AR blockade reverses ICI resistance

**Fig. 5:**
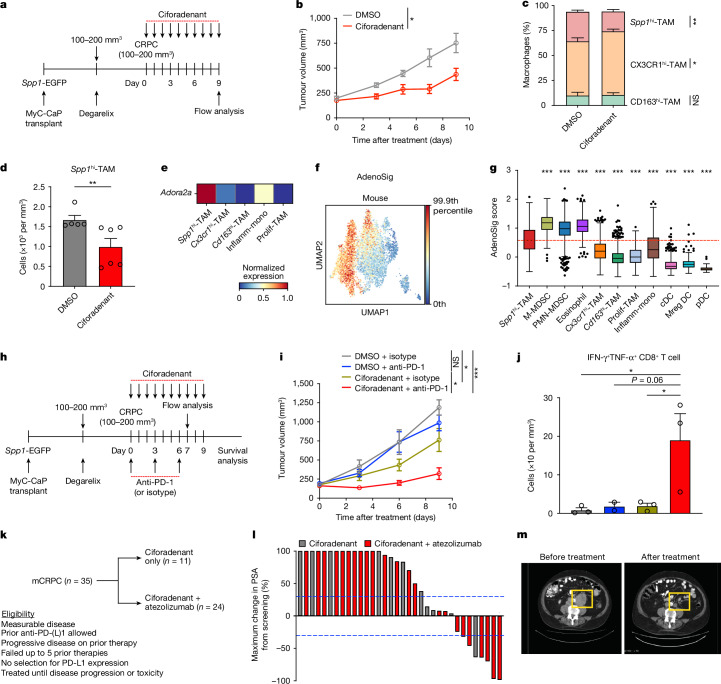
Inhibition of adenosine signalling diminishes the abundance of *Spp1*^hi^-TAMs and enhances the responsiveness of CRPC to PD-1 blockade in vivo. **a**, Schematic depicting the dosing schedule for ciforadenant (10 mg kg^−1^) or DMSO in CRPC mice. **b**, Cumulative CRPC growth after ciforadenant (*n* = 6) or DMSO (*n* = 5) treatment, compiled from *n* = 2 experiments; symbols show mean ± s.e.m. **c**,**d**, Quantification of macrophage subset frequency (**c**) and *Spp1*^hi^-TAM numbers (**d**) in CRPC treated with ciforadenant or DMSO from the same experiments as **b**; bars show mean + s.e.m.; symbols represent individual mice. **e**, Heatmap of normalized *Adora2a* expression (A2AR encoding) in macrophages and monocytes from mouse prostate cancer. **f**,**g**, UMAP (**f**) and bar plots (**g**) showing AdenoSig scores among myeloid cells in mouse prostate cancer (*n* = 6,397 myeloid cells; *P* < 0.001 for *Spp1*^hi^-TAM versus other subsets). Boxes denote IQR, and bars denote 25% − 1.5 × IQR and 75% + 1.5 × IQR, with outliers exceeding 1.5 × IQR. The red dashed line shows the median score for *Spp1*^hi^-TAMs. **h**, Schematic of ciforadenant (10 mg kg^−1^) treatment with and without anti-PD-1 (400 μg) treatment in CRPC mice. **i**, Cumulative CRPC growth after the treatments in **h**, compiled from *n* = 3 experiments; symbols represent mean ± s.e.m. DMSO + isotype, *n* = 7; DMSO + anti-PD-1, *n* = 6; ciforadenant + isotype, *n* = 7; ciforadenant + anti-PD-1, *n* = 6. **j**, Density of polyfunctional (IFN-γ^+^TNF-α^+^) CD8^+^ T cells in CRPC after the treatments in **h**. Each group is represented using the same colour scheme as in **i**. Bars show mean + s.e.m. from *n* = 3 experiments; symbols represent individual mice. **k**, Schematic showing the dosing schedule for ciforadenant (100 mg twice a day for 28 days) with or without atezolizumab (840 mg, once every two weeks) in patients with mCRPC. **l**, Waterfall plot of maximum prostate-specific antigen (PSA) change from screening in patients treated with ciforadenant either alone (grey) or with atezolizumab (red). **m**, Computed-tomography images showing tumour reduction in a clinical responder with measurable disease after the combination treatment. Statistical significance was determined by two-sided unpaired Student’s *t*-tests (**b**,**c**), a two-sided Mann–Whitney test (**d**), a Kruskal–Wallis test with Dunn’s correction (**g**) and an ordinary one-way ANOVA with Sidak correction (**i**,**j**); *P < 0.05, **P < 0.01, ***P < 0.001; NS, not significant. Source Data

**Extended Data Fig. 8:**
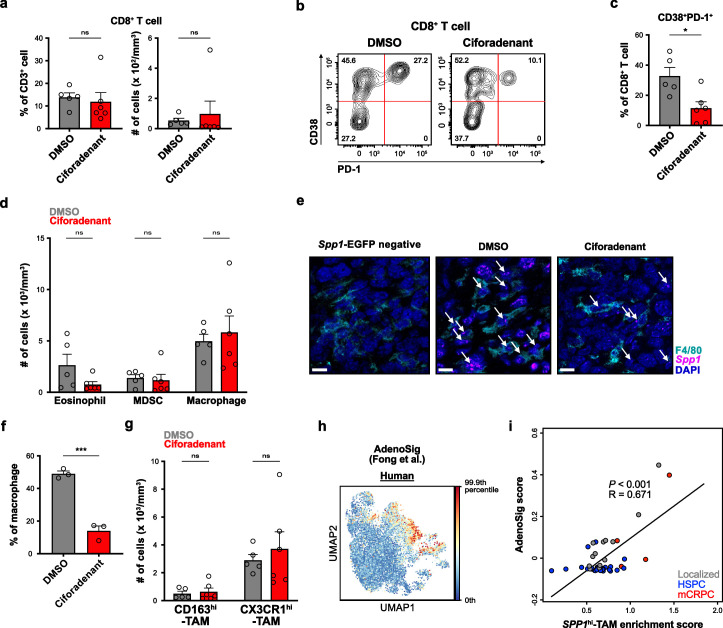
Evaluation of the lymphoid and myeloid compartments in CRPC treated with ciforadenant or DMSO control. (a) Quantification of the frequency (left) or number (right) of CD8^+^ T cells in CRPC developed in mice treated with ciforadenant (10 mg/kg; n = 6) or DMSO vehicle control (n = 5) from the same experiments as in Fig. 5b. (b-c) (b) Representative flow cytometry plots and (c) quantification of the frequency of CD8^+^ T cells exhibiting exhaustion states (CD38^+^PD-1^+^) from the same experiments as in Fig. 5b (*P* = 0.01). (d) Quantification of the number of the indicated major myeloid subsets from the same experiments as in Fig. 5b. (e) Representative immunofluorescent images of *Spp1*^hi^-TAMs in CRPC developed in either *Spp1*-EGFP negative (left) or positive mice treated with either DMSO (middle) or ciforadenant (right). Immunostaining for F4/80 (turquoise), *Spp1* (magenta), and DAPI (blue) is shown. Scale bars, 10 μm. White arrows indicate *Spp1*^hi^-TAMs. (f) Quantification of the frequency of *Spp1*^hi^-TAMs in CRPC developed in mice treated with ciforadenant or DMSO vehicle control (*P* < 0.001). (g) Quantification of the number of the indicated macrophage subsets in CRPC developed in mice treated with ciforadenant or DMSO vehicle control from the same experiments as in Fig. 5b. (h) UMAP plot showing enrichment scores for the “AdenoSig” gene signatures across myeloid cells in human prostate cancer. (i) Plot depicts the correlations between enrichment scores for the gene signatures “*SPP1*^hi^-TAMs” and “AdenoSig” in patient samples. Localized disease, HSPC, and mCRPC are in gray, blue, and red, respectively. The best-fit line is displayed, and individual patient samples are represented by circles. Bars represent the mean + SEM throughout this figure; symbols represent individual mice. Statistical significance was determined by (a, c, d, f, g) two-sided unpaired Student’s t-tests, and (i) a simple linear regression analysis; *P*-values: *<0.05, ***<0.001. ns, not significant.

**Extended Data Fig. 9:**
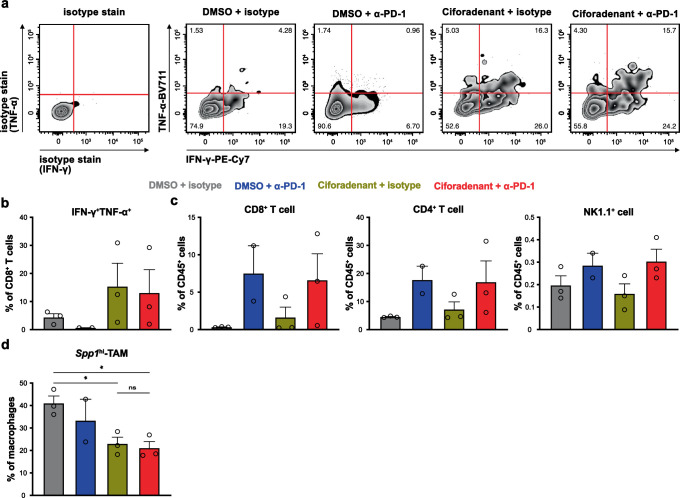
Assessment of lymphoid and myeloid compartments in CRPC following treatment with ciforadenant +/− anti-PD-1 antibody. (a-b) (a) Representative flow cytometry plots and (b) quantification showing an increase in the frequency of polyfunctional (IFN-γ^+^TNF-α^+^) CD8^+^ T cells following ciforadenant treatment with or without anti-PD-1 antibody, from the same experiments as in Fig. 5j. (c-d) Quantification of the frequency of (c) CD4^+^ T cells, CD8^+^ T cells, NK cells, and (d) *Spp1*^hi^-TAMs in CRPC developed in *Spp1*-EGFP mice treated with ciforadenant +/− anti-PD-1 antibody. Treatment groups are indicated as follows: DMSO + isotype antibody (gray; n = 3), DMSO + anti-PD-1 antibody (blue; n = 2), ciforadenant + isotype antibody (olive green; n = 3), and ciforadenant + anti-PD-1 antibody (red; n = 3) (*P* = 0.049, 0.03, and 0.99 for comparisons between DMSO + isotype vs. ciforadenant + isotype, DMSO + isotype vs. ciforadenant + anti-PD-1, and ciforadenant + isotype vs. ciforadenant + anti-PD-1, respectively). Bars represent the mean + SEM throughout this figure; symbols represent individual mice. Statistical significance was determined by (d) ordinary one-way ANOVA with the Sidak correction; *P*-values: *<0.05. ns, not significant.

**Extended Data Fig. 10:**
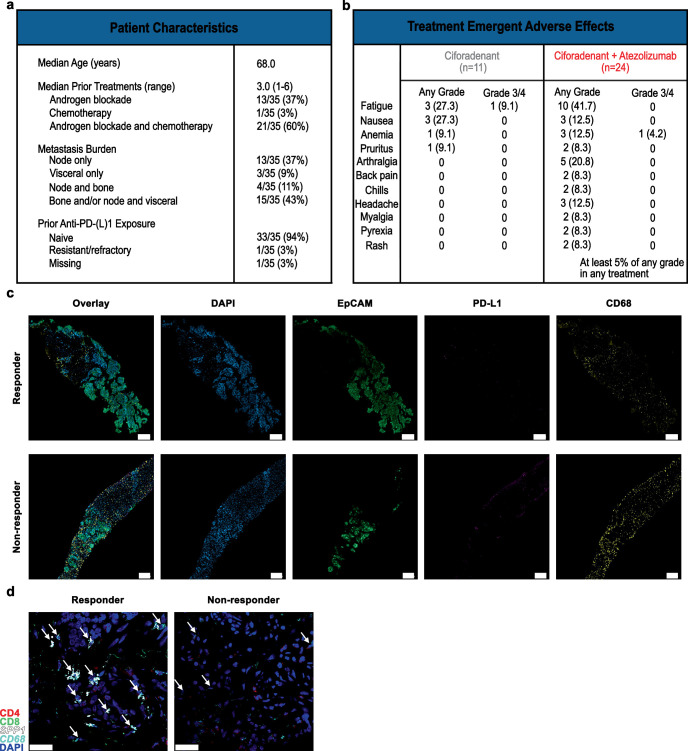
Clinical characteristics of the trial evaluating adenosine receptor blockade with ciforadenant +/− PD-L1 blockade using atezolizumab in mCRPC patients, and assessment of biopsies from responders and non-responders. (a) Patient characteristics from the clinical trial as shown in Fig. 5k. (b) Treatment emergent adverse effects observed in the patients. (c) Representative immunofluorescent images of baseline biopsy samples from responders and non-responders in the clinical trial. Immunostaining for EpCAM (green), PD-L1 (magenta), CD68 (yellow), and DAPI (blue) is presented. Scale bars, 200 μm. (d) Representative images of *SPP1*^hi^-TAMs in the same samples as in (c). Immunostaining for CD4 (red), CD8 (light green), *SPP1* transcripts (white), *CD68* transcripts (turquoise), and DAPI (blue) is presented. Scale bars, 30 μm. (c) and (d) represent data from one responder and two non-responders, which were the only samples available from this trial.

Considering that adenosine signalling probably underlies immunosuppression mediated by *SPP1*^hi^-TAMs, we then examined whether treating mice bearing CRPC with ciforadenant could alter the antitumour responses in vivo (Fig. 5a). Consistent with previous studies on mice with different cancer types, such as MC38 and B16 (refs. ^51,52^), blockade of A2ARs led to a significant reduction in CRPC growth, potentially resulting from a significantly lower frequency and number of exhausted CD8^+^ T cells (CD38^+^PD-1^+^; Extended Data Fig. 8a–c). Notably, evaluation of myeloid composition revealed alterations in the myeloid compartment. Although the overall number of the main myeloid populations remained largely unchanged (Extended Data Fig. 8d), the inhibition of A2ARs resulted in a significant decrease in both the frequency and number of *Spp1*^hi^-TAMs (Fig. 5c,d and Extended Data Fig. 8e,f), with no significant effect on other TAMs (Extended Data Fig. 8g). Further analyses of our mouse prostate cancer scRNA-seq dataset revealed elevated transcript levels of *Adora2a* (Fig. 5e) and higher enrichment scores for an adenosine gene signature expression (AdenoSig)^53^ (Fig. 5f,g), obtained by using a collection of genes with significantly induced expression on adenosine agonists in *Spp1*^hi^-TAMs compared with other macrophages and monocytes. Consistent with this, we identified enriched AdenoSig scores in *SPP1*^hi^-TAMs and observed a significant correlation between the enrichment scores for *SPP1*^hi^-TAM abundance and AdenoSig (Extended Data Fig. 8h,i) in humans. These findings indicate that adenosine signalling could be crucial for the abundance of *Spp1*^hi^-TAMs in CRPC. Given the significant decreases in the abundance of exhausted CD8^+^ T cells and *Spp1*^hi^-TAMs following treatment with ciforadenant, we proposed that A2AR blockade could augment the efficacy of ICIs. To test this possibility, we administered ciforadenant to mice bearing CRPC in combination with anti-PD-1, which showed limited therapeutic effectiveness as a monotherapy^27,38^ (Extended Data Fig. 5d), or relevant isotype-matched control antibodies (Fig. 5h). Notably, consistent with previous studies using different cancer types, including B16 and AT-3 (refs. ^54,55^), the dual blockade of A2ARs and PD-1 resulted in a significantly greater reduction in tumour growth than did monotherapies (Fig. 5i), indicating that A2AR inhibition contributes to enhancing the efficacy of ICIs in CRPC. Evaluation of the lymphoid compartment revealed that ciforadenant increased the frequency of polyfunctional CD8^+^ T cells, whereas PD-1 blockade enhanced the infiltration of T cells and NK cells into tumours (Extended Data Fig. 9a–c). Importantly, in line with previous studies^54,55^, combining PD-1 blockade with A2AR inhibition from ciforadenant increased the density of polyfunctional CD8^+^ T cells relative to monotherapies (Fig. 5j), indicating a mechanism underlying enhanced antitumour activity from combination therapy. Our assessment of myeloid cells indicated that the frequency of *Spp1*^hi^-TAMs was not further reduced by combining PD-1 blockade with ciforadenant compared with ciforadenant alone (Extended Data Fig. 9d). These findings highlight that the increased abundance of activated CD8^+^ T cells has a key role in the enhanced antitumour responses observed with combination therapy. On the basis of these findings, we evaluated the effect of A2AR blockade on immunotherapeutic resistance in humans in a phase 1 clinical trial (NCT02655822). Ciforadenant was administered to patients with mCRPC after failing at least one next-generation androgen blockade (Extended Data Fig. 10a), either alone (*n* = 11) or in combination with atezolizumab (*n* = 24) (Fig. 5k). The dosing frequency for this trial was as follows: ciforadenant, 100 mg twice a day for 28 days; and atezolizumab, 840 mg once every 2 weeks. This treatment was well tolerated, with very low frequencies of major side effects (Extended Data Fig. 10b). Of the 24 patients, 6 (25%) had a decrease in prostate-specific antigen levels from the baseline of 30% or more (Extended Data Table 1), which was confirmed 4 weeks later, and tumour regression was observed in some patients with measurable disease (Fig. 5l,m). Mutational analyses of two responders showed that their tumours were microsatellite stable and lacked CDK12 alterations, which could have sensitized the cancer to the ICI treatment (Extended Data Table 1). Moreover, analysis of biopsies from one responder and two non-responders demonstrated limited PD-L1 expression in all tissues (Extended Data Fig. 10c). Importantly, the responder had a higher prevalence of *SPP1*^hi^-TAMs at baseline (Extended Data Fig. 10d). These findings indicate that baseline *SPP1*^hi^-TAM abundance may serve as a potential biomarker for therapeutic efficacy, although further investigation with a larger cohort is warranted. Taken together, therapeutic interventions targeting adenosine signalling could represent a potential strategy to sensitize mCRPC to ICI treatments.

## Discussion

Although the establishment of an immunosuppressive niche by tumour-infiltrating myeloid cells in the prostate TME is well recognized, especially after ADT^3,4,15–18^, efforts to target these cells to enhance anti-tumour responses in patients with mCRPC have faced problems. For example, using CSF1R inhibition to target macrophages (the predominant myeloid subset in tumours) have shown limited antitumour activity^19,20^. Efforts to deplete intratumoral neutrophils or MDSCs by inhibiting myeloid chemotaxis through CXCR2 blockade have shown a reduction in therapeutic resistance to ADT^30^. Thus, a detailed understanding of the heterogeneity of myeloid cells, as well as the key regulators that govern myeloid programs within tumours^56^, is crucial for addressing therapeutic resistance. Despite numerous studies on the prostate TME at single-cell resolution^25–28^, there is no comprehensive single-cell atlas of myeloid cells across the disease continuum. Our findings, which are derived from patient biopsies and relevant mouse models, highlight the heterogeneity of myeloid cells in prostate cancer. Notably, *SPP1*^hi^-TAMs, which were identified as a prevalent myeloid subset in advanced disease, express diminished *CSF1R* transcript levels, providing an explanation for the lack of clinical efficacy in targeting this receptor. Comparative transcriptome analyses between humans and mice identified analogous *Spp1*^hi^-TAMs in mouse CRPC in an unbiased manner, which demonstrates their resistance to anti-CSF1R treatment in vivo. During the development of CRPC in mice, *Spp1*^hi^-TAMs significantly decrease in frequency, although their cell number remains consistent, mainly as the result of a substantial increase in both the frequency and number of infiltrating *CX3CR1*^hi^-TAMs (Extended Data Fig. 4c,d). Conversely, in human mCRPC, the *SPP1*^hi^-TAM fraction increases significantly as the disease progress (Fig. 1c,d), becoming at least as prevalent as *CX3CR1*^hi^-TAMs, if not more so. Given the diminished *CSF1R* transcript levels in *SPP1*^hi^-TAMs (Fig. 1h and Extended Data Fig. 2g), this could partly explain the clinical ineffectiveness of CSF1R antagonism in human patients with cancer compared with pre-clinical models. It will therefore be important to evaluate whether targeting immune inhibitory signals provided by *SPP1*^hi^-TAMs, along with CSF1R blockade, will elicit antitumour responses and augment the efficacy of ICIs in patients.

Although *SPP1*^hi^-TAMs have been identified in other cancer types^21,42,57^, their roles in prostate cancer progression, particularly as drivers of immunotherapy resistance and through molecular mechanisms, have not been functionally investigated. In this study, we demonstrate that *Spp1*^hi^-TAMs induce resistance to ICIs through adoptive transfer into CRPC. Our single-cell transcriptional analysis of human and mouse prostate cancers identified adenosine signalling as one of the main pathways preferentially activated in *SPP1*^hi^-TAMs, and functional assays subsequently confirmed that these macrophages contribute to extracellular adenosine accumulation in the prostate TME. Disrupting adenosine signalling, through either A2AR inhibitors or CD73-targeting antibodies, significantly reduced *Spp1*^hi^-TAM-mediated suppression of CD8^+^ T cells in co-culture, indicating that adenosine-associated signals are potential immunotherapeutic targets. However, the observation that T cell proliferation was not fully restored despite A2AR or CD73 blockade implies that there are more mechanisms underlying *Spp1*^hi^-TAM-mediated immunosuppression. Further transcriptional and functional analyses indicated that IL-1R signalling could have a role in *SPP1*^hi^-TAM-mediated resistance (Extended Data Fig. 7d–g), in line with previous findings^50^. Moreover, alterations in metabolic processes, including dysregulated lipid metabolism, potentially mediated by upregulated *Trem2*, have also been identified in *SPP1*^hi^-TAMs, indicating a link to prostate cancer growth, invasiveness and therapeutic resistance^58^. Therefore, examining T cell modulation by *SPP1*^hi^-TAMs in vivo through various approaches, including spatial transcriptomics, will be a focus of future studies. Moreover, a deeper understanding of the immunosuppressive mechanisms used by these macrophages will be crucial in identifying further therapeutic targets to enhance efficacy.

In our clinical trial, we observed that patients with mCRPC may benefit more from the combination of ciforadenant and atezolizumab than from atezolizumab alone^2^. Although promising, results from another study of AZD4635 (another A2AR antagonist) combined with durvalumab and cabazitaxel in patients with mCRPC (AARDVARC) failed to show a benefit with A2AR antagonism^59^. The discrepancy between the trials could have resulted from chemotherapy inclusion, the use of a different A2AR antagonist and/or patient selection. Despite the improved clinical activity observed with the combination treatment, antitumour responses were evident in only one of four patients in our trial. Moreover, although combined A2AR and PD-1 blockade significantly prolonged survival in a mouse model of CRPC, the mice did eventually die from the disease. These findings indicate that other immunosuppressive elements within the TME would need to be targeted simultaneously for even more effective immunotherapeutic intervention. Previous studies have identified various signals that contribute to the immunosuppressive nature of the prostate TME. For example, prostate cancer-associated fibroblasts promote immunosuppression on T cells by the release of transforming growth factor-β^60^ or by recruiting suppressive myeloid cells through the CCL2 and CXCL12 pathways^25^. Furthermore, castration-induced CXCL1, CXCL2 and IL-8 from prostate cancer cells mediate myeloid infiltration, particularly of MDSCs, resulting in an immunosuppressive TME^17,18^. Thus, a better understanding of further immunosuppressive TME elements beyond myeloid cells and their role in resistance to ICIs could reveal other therapeutic opportunities in mCRPC and provide strategies for patient selection.

Collectively, the data in this study demonstrate that *SPP1*^hi^-TAMs become increasingly abundant during prostate cancer progression and promote immunotherapeutic resistance through adenosine-mediated immunosuppression. Inhibition of A2AR delays CRPC progression and improves the responsiveness of tumour cells to PD-1 blockade. Moreover, our clinical trial shows that a subset of patients with mCRPC may benefit from ciforadenant plus atezolizumab instead of monotherapies. The abundance of *SPP1*^hi^-TAMs could serve as a biomarker to select for patients in future trials. Inhibiting adenosine signalling, as well as targeting chemokine or growth-factor receptor pathways, could further enhance the efficacy of immunotherapy in this and perhaps other refractory cancers.

## Methods

### scRNA-seq of samples derived from patients with prostate cancer

Tumour tissues were obtained from baseline biopsies of patients participating in clinical trials for localized prostate cancer (NCT03821246), de novo oligometastatic prostate cancer (NCT03007732) and metastatic mCRPC (NCT03248570). Viable cryopreserved tumour tissue samples were digested in Roswell Park Memorial Institute (RPMI) medium containing Collagenase I and II (0.1 mg ml^−1^, Thermo Fisher Scientific) and DNase I (Thermo Fisher Scientific), minced and then subjected to 1 h digestion using the gentleMACS system (Miltenyi Biotec). Live cell isolation was done using MACS LS columns (Miltenyi Biotec). The 10x Genomics Chromium Controller was used to generate GEM bead emulsions using the Single Cell 5′ Library & Gel Bead Kit (10x Genomics), followed by cDNA synthesis and amplification, and subsequent library preparation steps using 10x Genomics kits. Library sequencing was done by the University of California, San Francisco (UCSF) Institute for Human Genetics core on a NovaSeq 6000 (Illumina), targeting a median read depth of 150,000 reads per cell for total gene expression libraries and 60,000 reads per cell for CITE-seq libraries. All antibodies were obtained from BioLegend unless otherwise indicated. This work was done with informed consent obtained from all human research participants, and the sample procurement and analysis were approved by the institutional review board committees at UCSF.

### Human scRNA-seq analysis

The raw data from 10× sequencing were processed using the Cell Ranger pipeline (v.3, Genome build, GRCh38). The raw gene-expression matrices were subjected to processing by CellBender (v.0.1.0)^61^ to eliminate ambient RNAs. The filtered gene-expression matrices then underwent doublet detection using the package DoubletDetection (10.5281/zenodo.2678041) with default parameters. The results were analysed through the SCANPY pipeline^62^. To ensure the retention of high-quality cells, the following filters were applied: first, cells with less than 10% mitochondrial genes were retained; second, the number of detected genes per cell was set between 100 and 2,500 genes; third, genes expressed in at least three cells were kept; and finally, platelets (PF4, unique molecular identifier (UMI) > 0), red blood cells (HBB, UMI > 1) and doublets were removed. The gene-expression matrix was log_2_-transformed with the addition of 1 and normalized to 10,000 counts per cell, followed by highly variable gene selection using default parameters with the SCANPY function. The resulting matrix was corrected by regressing out total UMI counts and mitochondria percentage, followed by scaling to a mean of 0 and a variance of 1. Principal component analysis was performed using the top 50 principal components, followed by sample-wise batch correction using the SCANPY-implemented Harmony^63^. Leiden clustering (default resolution = 1.0) and UMAP plotting were performed, with a resolution of 1.0 applied for both T cell and myeloid cell clustering. Differential expression analysis identified the top-ranked genes that were upregulated in each individual cluster relative to the combination of all other cells, as determined by the SCANPY function tl.rank_genes_groups. Annotation of each unbiased population was achieved through manual inspection of the top-ranked genes of each cluster. Analysis of cell density on the UMAP was carried out using the SCANPY function tl.embedding_density, and boxplots were generated to represent cell population frequencies for each cell type. Gene scores were computed using the SCANPY function tl.score_genes with curated gene lists provided. To calculate gene scores at the sample level, scores were computed for each cell and subsequently combined at the sample level by using the median score of cells within a given sample.

### Mice

FVB/NJ and C57BL/6J male mice (from the Jackson Laboratory) were used in the experiments at 6–10 weeks of age. The STOCK Tg(Spp1-EGFP)PD43Gsat/Mmucd (*Spp1*-EGFP)^64^ mouse strain was sourced from the Mutant Mouse Resource & Research Centers at the University of California, Davis. All mice were housed in a pathogen-free facility under standardized environmental conditions, including a controlled 12 h:12 h light:dark cycle, humidity of 30–70% and a temperature range of 20–26 °C. For experiments, a total of 1 × 10^6^ cells (murine prostate cancer cell line MyC-CaP (CRL3255, American Type Culture Collection (ATCC)) or TRAMP-C2 (CRL-2731, ATCC)) were resuspended in sterile PBS and transplanted subcutaneously in the right flank of either FVB/NJ or C57BL/6J mice, respectively. The identities of MyC-CaP and TRAMP-C2 were authenticated using the Mouse Cell STR Profiling Service (137-XV, ATCC), and mycoplasma contamination was tested before each injection using a mycoplasma PCR detection kit (G238, abm). Sample size was determined using preliminary data and previous publications to ensure reproducibility of the experiment. Tumour volume = (*L* × *W* × *W*)/2 (mm^3^), with length (*L*) and width (*W*) being the longest diameter and shortest diameter, respectively. All experimental procedures were approved by the Institutional Animal Care and Use Committee at UCSF.

The model for CRPC was established by subcutaneously engrafting 1 × 10^6^ MyC-CaP cells into the right flank of male FVB/NJ mice 6–10 weeks old. When the tumour size reached 100–200 mm^3^, each mouse was injected subcutaneously with 1.875 mg degarelix (Firmagon) in 100 μl PBS, followed by a maintenance dose of 0.625 mg degarelix in 100 μl PBS every 28–30 days to induce CRPC. The development of CRPC was defined as a tumour volume that regressed after degarelix treatment and then grew back to 100–200 mm^3^. Subsequently, the mice were randomized and treated with the indicated antibodies and/or inhibitors.

### Cell line culture

MyC-CaP and TRAMP-C2 cells were cultured in complete DMEM medium comprising Dulbecco’s Modified Eagle Medium supplemented with 10% fetal bovine serum (FBS, Omega Scientific) and 1× penicillin/streptomycin (10,000 ml streptomycin sulfate and 10,000 units ml^−1^ penicillin G). All reagents were obtained from the UCSF Cell Culture Facility, unless otherwise indicated.

### Flow cytometric analysis

Mouse organs were collected and processed as follows. Spleens were mechanically dissociated with FACS wash buffer (PBS supplemented with 2% (v/v) FBS and 0.5 mM EDTA (Teknova)). Tumours were sequentially digested three times with 12 ml of a cocktail of 2 mg ml^−1^ (w/v) collagenase type IV and 100 Kunitz units per ml DNase I (both from Sigma-Aldrich) for 12 min for each digest. All single-cell suspensions were filtered using 70-μm filters (Fisher Scientific) and subjected to red-blood-cell lysis using ACK Lysing Buffer (Quality Biological). Cells were immunostained by incubating at 4 °C for 30 min with the fluorescently labelled antibodies below (all antibodies were purchased from BioLegend unless otherwise indicated). After staining, cells were washed once or twice in FWB and resuspended in FWB or FWB containing 1 μg ml^−1^ propidium iodide (PI, BioLegend) to assess viability. All flow cytometric data were acquired using an LSRFortessa X-50 flow cytometer operated with FACSDiva software (BD Biosciences). Post-acquisition data analysis was performed using FlowJo (v.10.10.0, Tree Star). All antibodies used in this study are commercially available and have been validated by the manufacturer or through published literature. On receipt, laboratory testing was conducted with known positive and negative controls to confirm the reliability of each antibody.

For mouse lymphoid staining, we used anti-mouse CD3-Brilliant Ultraviolet 395 (563565, Clone 145-2C11, BD Biosciences, 1:200), CD4-Brilliant Violet 711 (100447, GK1.5, 1:200), CD8-Brilliant Ultraviolet 805 (612898, 53-6.7, BD Biosciences, 1:200), NK-1.1-Alexa Fluor 647 (108719, PK136, 1:200), CD38-PE/Cyanine7 (102717, 90, 1:200), CD39-Brilliant Violet 421 (567105, Y23-1185, BD Biosciences, 1:200), CD45-Brilliant Violet 785 (103149, 30-F11, 1:200), CD279 (PD-1)-PE/Dazzle 594(109115, RMP1-30, 1:200) antibodies were used. For mouse myeloid staining, anti-mouse CD11b-Brilliant Violet 605 (101257, M1/70, 1:200), CD39-Brilliant Violet 421 (567105, Y23-1185, BD Biosciences, 1:200), CD73-PE (12-0731-82, eBioTY/11.8 (TY/11.8), Invitrogen, 1:200), CX3CR1-PE/Cyanine7 (149015, SA011F11, 1:200), F4/80-Alexa Fluor 647 (565853, T45-2342, BD Biosciences, 1:200), I-A/I-E-Alexa Fluor 700 (107621, M5/114.15.2, 1:200), Ly-6G-APC/Cyanine7 (127623, 1A8, 1:200), Podoplanin-PerCP/Cyanine5.5 (127421, 8.1.1, 1:200), Siglec-F-Brilliant Violet 421 or Brilliant Ultraviolet 395 (562681 or 740280, E50-2440, BD Biosciences, 1:200) antibodies. The relevant isotype-matched antibodies (eBRG1, RTK2758, RTK4530 and SHG-1) were used as controls.

For intracellular immunostaining of proteins, single-cell suspensions were labelled with LIVE/DEAD Fixable Aqua Dead Cell Stain Kit (L34957, Invitrogen, 1:1,000) and then treated with eBioscience Foxp3/Transcription Factor Staining Buffer Set (Invitrogen), according to the manufacturer’s protocol designed for intracellular (cytoplasmic) proteins. Cells were then stained with fluorescently labelled antibodies against anti-mouse CD3; Brilliant Ultraviolet 395 (563565, 145-2C11, BD Biosciences, 1:200), CD8-Brilliant Ultraviolet 805 (612898, 53-6.7, BD Biosciences, 1:200), CD11b-Brilliant Violet 605 (101257, M1/70, 1:200), CD45-Brilliant Violet 785 (103149, 30-F11, 1:200), IFN-γ-PE/Cy7 (505825, XMG1.2, 1:100), and TNF-α-Brilliant Violet 421 (506327, MP6-XT22, 1:100). The relevant isotype-matched antibodies (RTK2071) were used as negative controls.

### In vitro co-culture of purified myeloid cells with T cells

Complete RPMI cell culture medium was made up of RPMI 1640 supplemented with 10% FBS (Omega Scientific), 1× β-mercaptoethanol (Gibco, 55 µM), 1× glutamine (29.2 g l^−1^ l-glutamine, 200 mM), 1 mM sodium pyruvate (11 g  l^−1^ sodium pyruvate), 1× MEM non-essential amino acids, 1× penicillin/streptomycin (10,000 μg ml^−1^ streptomycin sulfate and 10,000 units ml^−1^ penicillin G). All reagents were obtained from the UCSF Cell Culture Facility, unless otherwise indicated.

For enrichment of mouse CD8^+^ T cells, single-cell suspensions of spleens from CRPC-bearing mice were labelled with BD Violet proliferation dye 450 (Fisher Scientific) and subsequently negatively enriched using the MojoSort Mouse CD8 T cell isolation kit, according to the manufacturer’s instructions. For isolation of specific myeloid subsets, single-cell suspensions from CRPC developed in *Spp1*-EGFP mice were incubated with the LIVE/DEAD Fixable Aqua Dead Cell Stain Kit (L34957, Invitrogen, 1:1,000), anti-mouse CD11b-Brilliant Violet 605 (101257, M1/70, 1:200), CX3CR1-PE/Cyanine7 (149015, SA011F11, 1:200), F4/80-Alexa Fluor 647 (565853, T45-2342, BD Biosciences, 1:200), I-A/I-E-Alexa Fluor 700 (107621, M5/114.15.2, 1:200), Ly-6G-APC/Cyanine7 (127623, 1A8, 1:200), Podoplanin-PerCP/Cyanine5.5 (127421, 8.1.1, 1:200) and Siglec-F-Brilliant Violet 421 (562681, BD Biosciences, 1:200) antibodies. After immunostaining, cells were washed twice in FWB and resuspended in FWB containing 1 μg ml^−1^ propidium iodide to assess viability. The cells of interest were FACS-purified using BD FACSAria Fusion operated with FACSDiva software (BD Biosciences).

To determine whether TAM cells, including *Spp1*^hi^-TAMs, CX3CR1^hi^-TAMs and CD163^hi^-TAMs, mediate immunosuppression, 1 × 10^4^ CD8^+^ T cells, labelled with BD Violet Proliferation Dye 450 (BDB562158, Fisher Scientific, 1:1,000) and stimulated with 1 × 10^4^ Dynabeads Mouse T-Activator CD3/CD28 (Gibco) were cultured in the presence or absence of purified myeloid subsets at a 1:1, 5:1 or 10:1 ratio, respectively, in 200 µl complete RMPI medium in round (U)-bottom 96-well plates at 37 °C, 5% CO_2_ for 3 days. T cell proliferation was assessed by flow cytometry.

To determine whether *Spp1*^hi^-TAMs suppress T cell activation, 1 × 10^4^ CD8^+^ T cells, labelled with BD Violet Proliferation Dye 450 (Fisher Scientific) and activated with 1 × 10^4^ Dynabeads Mouse T-Activator CD3/CD28 (Gibco), were cultured with or without purified *Spp1*^hi^-TAMs at a 1:1 ratio in 200 µl complete RMPI medium in round (U)-bottom 96-well plates at 37 °C, 5% CO_2_ for 3 days. The cells were subsequently restimulated with 1× eBioscience Cell Stimulation Cocktail (plus protein transport inhibitors, Invitrogen) for 5 h. After washing, cells were stained for intracellular immunostaining of proteins. T cell activation was assessed by flow cytometry.

To determine whether *Spp1*^hi^-TAM-mediated T cell suppression requires adenosine signalling, 1 × 10^4^ CD8^+^ T cells, labelled with BD Violet Proliferation Dye 450 (BDB562158, Fisher Scientific, 1:1,000) and activated with 1 × 10^4^ Dynabeads Mouse T-Activator CD3/CD28 (Gibco), were cultured with or without purified *Spp1*^hi^-TAMs at a 1:1 ratio in 200 µl complete RMPI medium in the presence of ciforadenant (10 μM, Corvus Pharmaceuticals) or *InVivo*MAb anti-mouse CD73 (10 μg ml^−1^; TY/23, BioXCell) in round (U)-bottom 96-well plates at 37 °C, 5% CO_2_ for 3 days. T cell proliferation was assessed by flow cytometry and compared with cells treated with the equivalent amount of DMSO or isotype-matched control antibodies (2A3, BioXCell).

To determine whether IL-1R signalling is involved in *Spp1*^hi^-TAM-mediated T cell suppression, 1 × 10^4^ CD8^+^ T cells, labelled with BD Violet Proliferation Dye 450 (BDB562158, Fisher Scientific, 1:1,000) and activated with 1 × 10^4^ Dynabeads Mouse T-Activator CD3/CD28 (Gibco), were cultured with or without purified *Spp1*^hi^-TAMs at a 1:1 ratio in 200 µl complete RMPI medium in the presence of either purified in vivo GOLD functional grade anti-mouse IL-1R (10 μg ml^−1^, JAMA-147) or the relevant isotype-matched control antibody (PIP, both from Leinco Technologies) in round (U)-bottom 96-well plates at 37 °C, 5% CO_2_ for 3 days. T cell proliferation was assessed by flow cytometry.

### Extracellular adenosine detection

*Spp1*^hi^-TAMs and MDSCs (both 1 × 10^5^ cells) were isolated from the same CRPC and plated in 250 µl complete RMPI medium in flat-bottom 48-well plates at 37 °C, 5% CO_2_. After 24 h, supernatants were collected and adenosine levels were measured using an adenosine assay kit (Fluoreometric, ab211094, Abcam) following the manufacturer’s protocol. Fluorescence was measured using a GluoMax plate reader (Promega; Ex/Em = 535/587 nm), and the concentration of accumulated extracellular adenosine was calculated by subtracting the baseline adenosine levels from medium without cells.

### Transwell assays

Transwell assays were performed as previously reported^65^. In brief, FACS-isolated *Spp1*^hi^-TAMs or enriched CD8^+^ T cells labelled with BD Violet Proliferation Dye 450 (BDB562158, Fisher Scientific, 1:1,000), from CRPC developed in mice or their spleens respectively, were plated into the top or bottom chambers of Transwell plates containing 6.5 mm cell culture inserts with 0.4 µm pore polyester membrane (CLS3379, Corning), as depicted in Extended Data Fig. 6e. In the top chamber of the inserts, 1 × 10^4^ CD8^+^ T cells alone or those stimulated with 1 × 10^4^ Dynabeads Mouse T-Activator CD3/CD28 (Gibco) were plated, and in the bottom chamber, 6 × 10^4^ *Spp1*^hi^-TAMs or 6 × 10^4^ CD8^+^ T cells with or without anti-CD3/28 stimulation at a 1:1 ratio were plated, each with 100 µl or 600 µl complete RMPI medium, respectively. After 3 days of culture, T cell proliferation in each chamber was assessed by flow cytometry.

### In vivo treatment of antibodies or inhibitors

To determine whether *Spp1*^hi^-TAMs are resistant to CSF1R blockade, mice were randomly divided into two groups when they developed CRPC (tumour volume of 100–200 mm^3^) and were administered intraperitoneally 1 mg anti-mouse CSF1R (AFS98, BioXCell) or the respective isotype-matched control (2A3, BioXCell) antibodies in 200 μl PBS. A maintenance dose of 0.5 mg in 200 μl PBS was given after 5 days. The myeloid composition was analysed by flow cytometry 2 days after the final injection.

For immune checkpoint inhibition, mice with established CRPC (tumour volume of 100–200 mm^3^) were randomly divided into four groups and subjected to intraperitoneal injection with these antibodies in 200 μl PBS every 3 days for a total of three injections: 200 μg anti-mouse CTLA-4 (24H2)^66^ alone; 400 μg anti-mouse PD-1 (17D2)^67^ alone; a combination of anti-mouse CTLA-4 and PD-1; or the respective IgG2a, κ isotype-matched control. Tumour burden was measured every 2–3 days after the initial injection until it reached 750 mm^3^, unless otherwise indicated.

To determine whether *Spp1*^hi^-TAMs contribute to resistance to ICIs in vivo, mice with developed CRPC (tumour volume of 100–200 mm^3^) were randomly divided into three groups. They were administered with: a combination of anti-mouse CTLA-4 and PD-1 in 200 μl PBS injected intraperitoneally along with intratumoral injection of 1 × 10^5^ *Spp1*^hi^-TAMs purified from digested CRPC (more than 350 mm^3^) of a mouse from the same cohort in 50 μl PBS; a combination of anti-mouse CTLA-4 and PD-1 in 200 μl PBS injected intraperitoneally along with 50 μl of PBS intratumorally; or the respective isotype-matched control antibody in 200 μl PBS injected intraperitoneally along with 50 μl PBS intratumorally. Antibodies were administered every 3 days for a total of three injections, and *Spp1*^hi^-TAMs were adoptively transferred every 5 days for a total of two injections. Tumour growth was measured every 2–3 days after the initial injection until it reached 750 mm^3^, unless otherwise indicated. The lymphoid composition was analysed by flow cytometry one day after the final injection.

For blockade of adenosine receptors (A2ARs), mice with established CRPC (tumour volume, 100–200 mm^3^) were randomly divided into two groups. Ciforadenant (10 mg per kg, Corvus Pharmaceuticals) or DMSO vehicle control (Sigma-Aldrich) in 200 μl of injection solution was administered once daily through oral gavage for 10 consecutive days. The injection solution consisted of 10% ciforadenant (or DMSO medium) and 90% corn oil (MedchemExpress). Tumour growth was measured every 2–3 days after the initial injection.

To determine whether A2AR blockade enhances immunotherapy efficacy, mice with established CRPC (tumour volume, 100–200 mm^3^) were randomly divided into two groups. Ciforadenant (10 mg per kg, Corvus Pharmaceuticals) or DMSO vehicle control (Sigma-Aldrich) in 200 μl of injection solution described above was administered once daily by oral gavage for 10 consecutive days. Simultaneously, mice were injected intraperitoneally with 400 µg anti-mouse PD-1 or the respective isotype-matched control antibodies in 200 µl PBS every 3 days for a total of three injections. Tumour growth was monitored every 2–3 days after the initial injection. The lymphoid and myeloid compositions were analysed by flow cytometry 1–2 h after the eighth injection of ciforadenant (1 day after the final anti-mouse PD-1 antibody injection).

All comparisons within experiments were carried out using age-matched mice (6–10 weeks old) engrafted with the same stock of MyC-CaP throughout the study.

### scRNA-seq of prostate cancer mouse samples

For the single-cell assessment of MyC-CaP, a cohort of FVB/NJ mice bearing MyC-CaP were injected subcutaneously with either degarelix (*n* = 3) or PBS (*n* = 3), as described above. HSPC or CRPC tissues were collected on reaching a tumour volume of more than 350 mm^3^. Tumours were processed and single-cell suspensions were prepared as described above. For the cell-surface protein staining, cells were incubated with CD45.1-Brilliant Violet 510 (A20) for 30 min at 4 °C. After immunostaining, cells were washed twice in FWB and resuspended in FWB containing 1 μg ml^−1^ propidium iodide for viability assessment. Cells were then sorted into CD45^+^ and CD45^−^ populations using FACSAria (BD Biosciences). Each population was transferred into separate 75 mm flow-cytometry tubes, centrifuged for 5 min at 250*g* at 4 °C and the supernatant was discarded. Cells were then resuspended in 100 μl Fc blocking buffer, consisting of 95 μl FWB + 5 μl of 0.5 mg ml^−1^ anti-mouse CD16/32 antibody (2.4G2, Cytek Biosciences), followed by a 10-minute incubation at 4 °C. CD45^+^ cells were subsequently stained directly with 2 μl of 0.05 mg ml^−1^ TotalSeq-C hashtag antibodies 2, 4 and 6 (M1/42, 30-F11) without washing, for 40 min at 4 °C. All sorted populations from each tissue were then pooled to yield a total of 1 × 10^6^ cells and these cells were stained with 100 μl of a cocktail of TotalSeq-C surface antibodies (CD11c (N418), CD163 (S15049I), F4/80 (BM8) and Ly-6G (1A8), each at a concentration of 2.5 μg ml^−1^) for 30 min at 4 °C. After staining, cells were washed with 1 ml complete RPMI medium and filtered through a 70 µm cell strainer. Cell viability and counting were reassessed and the volumes were adjusted for 10x chromium chip input at a concentration of 1.29 × 10^6^ cells per ml. For scRNA-seq of TRAMP-C2, single-cell suspensions were initially labelled with LIVE/DEAD Fixable Dead Cell Stain Kit (Near-IR; Invitrogen) for 10 min at 4 °C. Subsequently, cells were stained with anti-mouse CD16/32 antibody (2.4G2) and CD45-Brilliant Violet 570 (30-F11) antibodies for 30 min on ice. After immunostaining, cells were washed with FWB and sorted into CD45^+^ and CD45^−^ populations using a FACSAria2 (BD Biosciences). Each sorted population was transferred into separate 75 mm flow-cytometry tubes, centrifuged for 5 min at 250*g* at 4 °C and the supernatant was discarded. Cells were then resuspended in 100 μl Fc blocking buffer as above, followed by a 10-minute incubation at 4 °C. Cells were then stained directly with 2 μl of 0.05 mg ml^−1^ TotalSeq-C hashtag antibodies 1 and 2 (M1/42, 30-F11) for 40 min at 4 °C without washing. Equal proportions of cells labelled with hashtags were pooled together, and three individual reactions, each containing a total of 6 × 10^4^ cells, were washed with 1 ml complete RPMI medium and filtered through a 70 µm cell strainer. After reassessing cell viability and counting, cell concentrations were adjusted to 1.29 × 10^6^ cells per ml for loading into the 10x chromium chip. A 10x Genomics chromium controller was used to create GEM bead emulsions using a Single Cell 5′ Library & Gel Bead Kit (10x Genomics), followed by synthesis and amplification of cDNA and subsequent library preparation steps using 10x Genomics kits. The UCSF Institute for Human Genetics core performed library sequencing on a NovaSeq 6000 (Illumina), targeting a median read depth of 150,000 reads per cell for total gene expression libraries and 60,000 reads per cell for CITE-seq libraries. All antibodies were obtained from BioLegend, unless otherwise indicated.

### Mouse scRNA-seq analysis

The raw data obtained from 10× sequencing were processed through the Cell Ranger pipeline (v.5, Genome build GRCm38). Subsequent steps in the analysis were the same as those used in human scRNA-seq until batch correction using Harmony, followed by Leiden clustering (resolution = 1.0) and UMAP plotting. A resolution of 1.0 was applied for myeloid-cell clustering. Differential expression analysis was done to identify the top-ranked genes upregulated in each individual cluster compared with the combination of all other cells. This analysis was done using the SCANPY function tl.rank_genes_groups. Annotation of each population was established through manual examination of the top-ranked genes in each cluster. To analyse cell density on the UMAP, the SCANPY function tl.embedding_density was used. Box plots were generated to visually represent cell population frequencies for each cell type. Gene scores were computed using the SCANPY function tl.score_genes with curated gene lists provided. Gene scores were computed using the SCANPY function tl.score_genes for each cell, using curated gene lists. To calculate similarity scores between myeloid subsets in humans and mice, a pseudo-bulk analysis was done to aggregate gene-expression data from the cellular level to the sample level. *Z*-scores were computed for each gene on the basis of cells in a given sample, and the mean was determined as the representative value for the sample. We then identified the shared genes in both human and mouse datasets, focusing on the top 50 genes within each subset.

### Immunostaining and microscopy

For immunostaining of *SPP1*^hi^-TAMs and CD4^+^ or CD8^+^ T cells in human tissues, in situ hybridization was done using RNAscope (Advanced Cell Diagnostics, ACDBio) on FFPE sections 4 μm thick from patients with either HSPC or mCRPC (NCT03007732, NCT03248570 and NCT02655822). Tissues were pretreated with target retrieval reagents and protease to improve target recovery according to the RNAscope Multiplex Fluorescent Reagent Kit v.2 assay protocol (323100, ACD Bio). Probes for human *SPP1* and *CD68* mRNA (420101-C2 and 560591-C4, respectively; ACDBio) were applied at a 1:50 dilution for 2 h at 40 °C. The probes were then hybridized with Opal 7-Color Manual IHC Kit (NEL811001KT, PerkinElmer) for the detection of *SPP1* and *CD68* transcripts using Opal 650 and Opal 690, respectively, at a dilution of 1:700. Immunofluorescence staining for human CD4 (MA-12259, 4B12, Invitrogen) and CD8 (ab60076, YTC182.20, abcam) was then done at a 1:100 dilution each. Targets were detected using Alexa Fluor 488-conjugated donkey anti-mouse IgG secondary antibody (ab150105, abcam) at a 1:100 dilution and Alexa Fluor 555-conjugated goat anti-rabbit IgG secondary antibody (4050-32, Southern Biotech) at a 1:100 dilution. Tissues were counterstained with 4′,6diamidino-2-phenylindole (DAPI, ACD Bio) and mounted with ProLong Gold Antifade Mountant (P36930, Invitrogen). Slides were imaged at 63× magnification using a Leica SP8 X white-light laser confocal microscope (Leica Microsystems) with multiple regions of interest from each specimen slide randomly selected for analysis. No staining was observed using negative control probes specific for the bacterial *dapB* gene (321831, ACD Bio) counterstained with Opal dyes, or with secondary antibodies alone on tonsil tissue.

Immunostaining of PD-L1 expression on EpCAM^+^ cells and CD68^+^ cells in human tissues was done on FFPE tissue sections 4 µm thick from responders and non-responders in clinical trial NCT02655822. This staining was done using a Ventana DISCOVERY ULTRA automated slide stainer and Ventana DISCOVERY ULTRA reagents (Roche Diagnostics), according to the manufacturer’s instructions (UCSF Protocol 3612), unless otherwise indicated. After deparaffinization, antigen retrieval was performed with cell conditioning 1 solution for 64 min at 97 °C. Primary antibodies for human CD68 (PG-M1; Agilent), PD-L1 and EpCAM (E1L3N and D9S3P, respectively, Cell Signaling Technology) were applied at 1:200, 1:100 and 1:50 dilutions for 32 min, respectively, at 36 °C. Goat Ig Block Ventana (760-6008) was applied for 4 min before the secondary antibodies (OmniMap anti-Ms for the anti-CD68 antibody and OmniMap anti-Rb for the anti-PD-L1 and anti-EpCAM antibodies) were incubated for 12 min. A stripping step between each primary was done with cell conditioning 2 solution at 97 °C for 8 min between primary antibodies. Endogenous peroxidase was inhibited using DISCOVERY Inhibitor RUO Ventana (760-4840) for 12 min. The CD68 was visualized using DISCOVERY Rhodamine 6G Kit Ventana (760-244), PD-L1 was visualized with DISCOVERY Cy5 Kit (760-238) and EpCAM was visualized with DISCOVERY FAM Kit (RUO) (760-243) for 8 min each. Finally, slides were counterstained with spectral DAPI (FP1490, Akoya) for 8 min. Slides were scanned using an AxioScan.Z1 in a whole-slide scanner (Zeiss) with a Plan-Apochromat 20×/0.8 M27 objective lens. Images were captured using an Orca-Flash 4.0 v.2 CMOS camera (Hamamatsu).

Immunostaining of mouse tissues was done on 5-μm acetone-fixed cryosections following standard protocols, as previously described^68^. Sections were immunostained with the following antibodies: anti-mouse F4/80-Alexa Fluor 647 (565853, T45-2342, BD Biosciences) at a 1:200 dilution, and *Spp1*-EGFP was amplified using chicken anti-GFP antibody (ab13970, abcam) at a 1:2,000 dilution, followed by donkey anti-chicken IgY(IgG)-DyLight 405 (703-475-155, Jackson ImmunoResearch) at a 1:500 dilution. After staining, slides were washed, stained with DAPI to detect nuclei and mounted with ProLong Gold Antifade Mountant (P36930, Invitrogen). Images were obtained on a Leica DMi8 microscope with a 63×/1.32 oil objective lens and a Leica DFC9000 GTC digital microscope camera, with LAS X software (v.3.5.7.23225). Images were processed using ImageJ (v.2.14.0/1.54 f) for fluorescent channel overlays and uniform exposure adjustment.

### Statistical analysis

Statistical analyses were done using Prism (v.10, GraphPad Software). Normality was determined with the D’Agostino & Pearson or Shapiro–Wilk tests, chosen according to sample size. Statistical significance was determined using two-sided unpaired Student’s *t*-tests for normally distributed data or the non-parametric Mann–Whitney test, two-sided paired Student’s *t*-tests, one sample *t*-tests, ordinary one-way ANOVA with Sidak correction for normally distributed data or the non-parametric Kruskal–Wallis test with Dunn’s correction, ordinary two-way ANOVA with Sidak correction, simple linear regression analyses, Wilcoxon tests with Benjamini–Hochberg correction, or log-rank tests, as indicated in the figure legends.

### Reporting summary

Further information on research design is available in the Nature Portfolio Reporting Summary linked to this article.

## Online content

Any methods, additional references, Nature Portfolio reporting summaries, source data, extended data, supplementary information, acknowledgements, peer review information; details of author contributions and competing interests; and statements of data and code availability are available at 10.1038/s41586-024-08290-3.

## Supplementary information

Reporting Summary

## Source data

Source Data Figs. 2–5

## Figures and Tables

**Extended Data Table 1:**
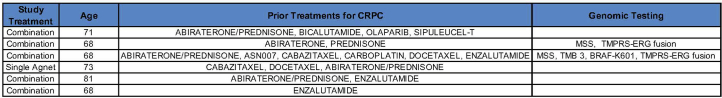
Details of prior treatments and genomic testing results for responders from the clinical trial evaluating ciforadenant and atezolizumab

## Data Availability

The data generated in this study are available in the article and its supplementary data files. The human and mouse scRNA-seq data have been deposited in the Gene Expression Omnibus database under accession number GSE274229. The human and mouse genome assemblies, GRCh38 and GRCm38, were obtained from the NIH National Library of Medicine website. Source data are provided with this paper.
