# Supplementary material for: Evolution of myeloid-mediated immunotherapy resistance in prostate cancer
Source: Nature. 2024 Dec 4;637(8048):1207–17. doi: 10.1038/s41586-024-08290-3 (PMC11779626; doi:10.1038/s41586-024-08290-3)
Supplement: Supplementary file 1 — Reporting Summary [file 41586_2024_8290_MOESM1_ESM.pdf]

nature portfolio

Corresponding author(s): Lawrence Fong

Last updated by author(s): Oct 22, 2024

Reporting Summary

Nature Portfolio wishes to improve the reproducibility of the work that we publish. This form provides structure for consistency and transparency in reporting. For further information on Nature Portfolio policies, see our Editorial Policies and the Editorial Policy Checklist.

Statistics

For all statistical analyses, confirm that the following items are present in the figure legend, table legend, main text, or Methods section.

n/a	Confirmed	
☐	☒	The exact sample size (*n*) for each experimental group/condition, given as a discrete number and unit of measurement
☐	☒	A statement on whether measurements were taken from distinct samples or whether the same sample was measured repeatedly
☐	☒	The statistical test(s) used AND whether they are one- or two-sided<br>*Only common tests should be described solely by name; describe more complex techniques in the Methods section.*
☒	☐	A description of all covariates tested
☐	☒	A description of any assumptions or corrections, such as tests of normality and adjustment for multiple comparisons
☐	☒	A full description of the statistical parameters including central tendency (e.g. means) or other basic estimates (e.g. regression coefficient) AND variation (e.g. standard deviation) or associated estimates of uncertainty (e.g. confidence intervals)
☒	☐	For null hypothesis testing, the test statistic (e.g. F, t, r) with confidence intervals, effect sizes, degrees of freedom and P value noted<br>*Give P values as exact values whenever suitable.*
☒	☐	For Bayesian analysis, information on the choice of priors and Markov chain Monte Carlo settings
☒	☐	For hierarchical and complex designs, identification of the appropriate level for tests and full reporting of outcomes
☒	☐	Estimates of effect sizes (e.g. Cohen's *d*, Pearson's *r*), indicating how they were calculated

Our web collection on statistics for biologists contains articles on many of the points above.

Software and code

Policy information about availability of computer code

Data collection	single-cell RNA-sequencing (a droplet-based 5’ 10x Genomics platform), flow cytometry: BD FACSDiva (multiple versions due to software updates)
Data analysis	flow cytometry: FlowJo (Tree Star, Inc.; v10, multiple versions due to software updates)<br>scRNA-seq: Cell Ranger pipeline (v3 for human samples and v5 for mouse samples), CellBender (v0.1.0), DoubletDetection (10.5281/zenodo.2678041), SCANPY packages (multiple versions due to software updates for each) including Harmony, Leiden, tl.rank_genes_group, tl.embedding_density, tl.score_genes, and Enrichr (https://maayanlab.cloud/Enrichr/)<br>statistical testing: Prism (GraphPad Software; v10, multiple versions due to software updates)

For manuscripts utilizing custom algorithms or software that are central to the research but not yet described in published literature, software must be made available to editors and reviewers. We strongly encourage code deposition in a community repository (e.g. GitHub). See the Nature Portfolio guidelines for submitting code & software for further information.

Data

Policy information about availability of data

All manuscripts must include a data availability statement. This statement should provide the following information, where applicable:

- Accession codes, unique identifiers, or web links for publicly available datasets
- A description of any restrictions on data availability
- For clinical datasets or third party data, please ensure that the statement adheres to our policy

The data generated in this study are available within the article and its supplementary data files, and source data are provided with this paper. The human and mouse scRNA-seq data analyzed in this study have been deposited in the Gene Expression Omnibus (GEO) database under accession number GSE274229. The human and mouse genome assemblies, GRCh38 and GRCm38, were obtained from the National Institutes of Health (NIH) National Library of Medicine website.

Research involving human participants, their data, or biological material

Policy information about studies with human participants or human data. See also policy information about sex, gender (identity/presentation), and sexual orientation and race, ethnicity and racism.

Reporting on sex and gender	Prostate cancer occurs only in individuals with male sex.
Reporting on race, ethnicity, or other socially relevant groupings	The researchers are not reporting on race, ethnicity, or other socially relevant groupings.
Population characteristics	The patients accrued to the clinical studies had the diagnosis of prostate cancer. Covariates include disease burden and prior lines of therapy for their prostate cancer.
Recruitment	De-identified primary patient samples were obtained from University of California San Francisco (UCSF).
Ethics oversight	Sample procurement and analysis were approved by the institutional review board committees at UCSF.

Note that full information on the approval of the study protocol must also be provided in the manuscript.

Field-specific reporting

Please select the one below that is the best fit for your research. If you are not sure, read the appropriate sections before making your selection.

☒ Life sciences ☐ Behavioural & social sciences ☐ Ecological, evolutionary & environmental sciences

For a reference copy of the document with all sections, see nature.com/documents/nr-reporting-summary-flat.pdf

Life sciences study design

All studies must disclose on these points even when the disclosure is negative.

Sample size	We performed a minimum of 2-3 experiments for each in vitro or in vivo study. The number of experiments was chosen based on our previous publications, including Pai et al., Immunity, to ensure experiment reproducibility. Moreover we considered relevant literature to confirm the proper utilization of materials, such as antibodies.
Data exclusions	No data were excluded from the analyses.
Replication	At least 2-3 successful independent biological replications were carried out for each experiment.
Randomization	We utilized age-matched FVB/NJ or Tg(Spp1-EGFP)PD43Gsat/Mmucd (Spp1-EGFP) male mice for the engraftment of MyC-CaP cells, controlling for variations in mouse background and age. Once CRPC was established, the mice were randomly divided into experimental and control groups. We utilized age-matched C57BL/6J male mice for the engraftment of TRAMP-C2 cells, controlling for variations in mouse background and age. Once tumor was established, the mice were randomly divided into experimental and control groups.<br>Patients were screened within oncology clinics at the different institutions and were consented to the clinical study if they met the inclusion criteria. Patients were sequentially accrued onto the ciforadenant monotherapy group and then onto the ciforadenant + atezolizumab group.
Blinding	The investigators were blinded to group allocation during data collection and data analysis.

Reporting for specific materials, systems and methods

We require information from authors about some types of materials, experimental systems and methods used in many studies. Here, indicate whether each material, system or method listed is relevant to your study. If you are not sure if a list item applies to your research, read the appropriate section before selecting a response.

Materials & experimental systems

n/a	Involved in the study
☐	☒ Antibodies
☐	☒ Eukaryotic cell lines
☒	☐ Palaeontology and archaeology
☐	☒ Animals and other organisms
☐	☒ Clinical data
☒	☐ Dual use research of concern
☒	☐ Plants

Methods

n/a	Involved in the study
☒	☐ ChIP-seq
☐	☒ Flow cytometry
☒	☐ MRI-based neuroimaging

Antibodies

Antibodies used

For mouse lymphoid staining, anti-mouse CD3-Brilliant Ultraviolet 395 (Cat #: 563565; Clone: 145-2C11; BD Biosciences; 1:200), CD4-Brilliant Violet 711 (100447; GK1.5; 1:200), CD8-Brilliant Ultraviolet 805 (612898; 53-6.7; BD Biosciences; 1:200), NK-1.1-Alexa Fluor 647 (108719; PK136;1:200), CD38-PE/Cyanine7 (102717; 90; 1:200), CD39-Brilliant Violet 421 (567105; Y23-1185; BD Biosciences; 1:200), CD45-Brilliant Violet 785 (103149; 30-F11; 1:200), CD279 (PD-1)-PE/Dazzle 594(109115; RMP1-30; 1:200) antibodies were used. For mouse myeloid staining, anti-mouse CD11b-Brilliant Violet 605 (101257; M1/70; 1:200), CD39-Brilliant Violet 421 (567105; Y23-1185; BD Biosciences; 1:200), CD73-PE (12-0731-82; eBioTY/11.8 (TY/11.8); Invitrogen; 1:200), CX3CR1-PE/Cyanine7 (149015; SA011F11; 1:200), F4/80-Alexa Fluor 647 (565853; T45-2342; BD Biosciences; 1:200), I-A/I-E-Alexa Fluor 700 (107621; M5/114.15.2; 1:200), Ly-6G-APC/Cyanine7 (127623; 1A8; 1:200), Podoplanin-PerCP/Cyanine5.5 (127421; 8.1.1; 1:200), Siglec-F-Brilliant Violet 421 or Brilliant Ultraviolet 395 (562681 or 740280; E50-2440; BD Biosciences; 1:200) antibodies were used. The relevant isotype antibodies (eBRG1, RTK2758, RTK4530 and SHG-1) were used as controls.

For intracellular immunostaining of proteins, single-cell suspensions were labeled with LIVE/DEAD Fixable Aqua Dead Cell Stain Kit (L34957; Invitrogen; 1:1000) and then treated with eBioscience™ Foxp3 / Transcription Factor Staining Buffer Set (Invitrogen) according to the manufacturer's protocol designed for intracellular (cytoplasmic) proteins. Cells were then stained with fluorescently labeled antibodies against anti-mouse CD3-Brilliant Ultraviolet 395 (563565; 145-2C11; BD Biosciences; 1:200), CD8-Brilliant Ultraviolet 805 (612898; 53-6.7; BD Biosciences; 1:200), CD11b-Brilliant Violet 605 (101257; M1/70; 1:200), CD45-Brilliant Violet 785 (103149; 30-F11; 1:200), IFN-γ-PE/Cy7 (505825; XMG1.2; 1:100), and TNF-α-Brilliant Violet 421 (506327; MP6-XT22; 1:100) antibodies. The relevant isotype antibodies (RTK2071) were used as negative controls.
For immunostaining of SPP1hi-TAMs and CD4+ or CD8+ T cells in human tissues, in situ hybridization was performed using RNAscope (Advanced Cell Diagnostics, Inc; ACDBio) on 4 µm thick FFPE sections from patients with either HSPC or mCRPC (NCT03007732, NCT03248570, and NCT02655822). Tissues were pre-treated with target retrieval reagents and protease to improve target recovery according to the RNAscope Multiplex Fluorescent Reagent Kit v2 Assay protocol (323100; ACD Bio). Probes for human SPP1 and CD68 mRNA (420101-C2 and 560591-C4, respectively; ACDBio,) were applied at a 1:50 dilution for 2 hours at 40°C. The probes were then hybridized with Opal 7-Color Manual IHC Kit (NEL811001KT; PerkinElmer) for the detection of SPP1 and CD68 transcripts using Opal 650 and Opal 690, respectively, at a 1:700 dilution. Immunofluorescence staining for human CD4 (MA-12259; 4B12; Invitrogen) and CD8 (ab60076; YTC182.20; abcam) was then carried out at a 1:100 dilution each. Targets were detected using Alexa Fluor 488-conjugated donkey anti-mouse IgG secondary antibody (ab150105; abcam) at a 1:100 dilution and Alexa Fluor 555-conjugated goat anti-rabbit IgG secondary antibody (4050-32; Southern Biotech) at a 1:100 dilution. Tissues were counterstained with 4',6diamidino-2-phenylindole (DAPI; ACD Bio) and mounted with ProLong Gold Antifade Mountant (P36930; Invitrogen). Slides were imaged at 63X magnification using a Leica SP8 X white light laser confocal microscope (Leica Microsystems), with multiple regions of interest from each specimen slide randomly selected for analysis. No staining was observed using negative control probes specific for the bacterial DapB gene (321831; ACD Bio) counterstained with Opal dyes, or with secondary antibodies alone on tonsil tissue.

Immunostaining of PD-L1 expression on EpCAM+ cells and CD68+ cells in human tissues was performed on 4 µm thick FFPE tissue sections from responders and non-responders in trial NCT02655822. This staining was conducted using a Ventana DISCOVERY ULTRA automated slide stainer and Ventana DISCOVERY ULTRA reagents (Roche Diagnostics), according to the manufacturer's instructions (UCSF Protocol 3612), unless otherwise indicated. After deparaffinization, antigen retrieval was performed with Cell Conditioning 1 (CC1) solution for 64 minutes at 97°C. Primary antibodies for human CD68 (PG-M1; Agilent), PD-L1 and EpCAM (E1L3N and D9S3P, respectively; Cell Signaling Technology) were applied at 1:200, 1:100, and 1:50 dilutions for 32 mins, respectively, at 36°C. Goat Ig Block Ventana (760-6008) was applied for 4 minutes before the secondary antibodies (OmniMap anti-Ms for the anti-CD68 antibody and OmniMap anti-Rb for the anti-PD-L1 and anti-EpCAM antibodies) were incubated for 12 minutes. A stripping step between each primary was performed with Cell Conditioning 2 (CC2) solution at 97°C for 8 minutes between primary antibodies. Endogenous peroxidase was inhibited using DISCOVERY Inhibitor RUO Ventana (760-4840) for 12 minutes. The CD68 was visualized using DISCOVERY Rhodamine 6G Kit Ventana (760-244), PD-L1 was visualized with DISCOVERY Cy5 Kit (760-238), and EpCAM was visualized with DISCOVERY FAM Kit (RUO) (760-243) for 8 minutes each. Finally, slides were counterstained with Spectral DAPI (FP1490; Akoya) for 8 minutes. Slides were scanned using an AxioScan.Z1 in a whole slide scanner (Zeiss) with a Plan-Apochromat 20x/0.8 M27 objective. Images were captured using an Orca-Flash 4.0 v2 CMOS camera (Hamamatsu).

Immunostaining of mouse tissues was performed on 5 µm acetone-fixed cryosections following standard protocols as previously described68. Sections were immunostained with the following antibodies: anti-mouse F4/80-Alexa Fluor 647 (565853; T45-2342, BD Biosciences) at a 1:200 dilution, and Spp1-EGFP was amplified using chicken anti-GFP antibody (ab13970, abcam) at a 1:2000 dilution, followed by donkey anti-chicken IgY(IgG)-DyLight 405 (703-475-155, Jackson ImmunoResearch) at a 1:500 dilution. The relevant isotype antibodies (Poly29108, BioLegend) were used as controls. After staining, slides were washed, stained with DAPI to detect nuclei, and mounted with ProLong Gold Antifade Mountant (P36930; Invitrogen). Images were obtained on a Leica DMi8 microscope with a 63x/1.32 oil objective and a Leica DFC9000 GTC digital microscope camera, with LAS X software (v3.5.7.23225). Images were processed using ImageJ (v2.14.0/1.54f) for fluorescent channel overlays and uniform exposure adjustment.

Validation	All antibodies used in this study are commercially available and have been validated by the manufacturer (BioLegend, BD Biosciences, abcam, and Invitrogen) or through published literature (Lyu et al., 2020, Blood, Lyu et al., 2023, Nat Commun, and Arias-Badia et al., 2024, Front Immunol). Upon receipt, laboratory testing was conducted with known positive and negative controls, such as tonsil tissues, to confirm the reliability of each antibody.

Eukaryotic cell lines

Policy information about cell lines and Sex and Gender in Research

Cell line source(s)	MyC-CaP and TRAMP-C2, sourced from ATCC, are epithelial-like cell lines isolated from the prostate of male mice with prostate cancer.
Authentication	Our MyC-CaP and TRAMP-C2 stocks were authenticated using the ATCC mouse STR Profile, confirming that "The submitted sample profile is an exact match for the following ATCC cell line(s) in the ATCC mouse STR database: CRL-3255 (MyC-CaP), CRL-2731 (TRAMP-C2)"
Mycoplasma contamination	We verified the absence of mycoplasma contamination prior to each engraftment.
Commonly misidentified lines (See ICLAC register)	N/A

Animals and other research organisms

Policy information about studies involving animals; ARRIVE guidelines recommended for reporting animal research, and Sex and Gender in Research

Laboratory animals	For engraftment of MyC-CaP cells, FVB/NJ and Tg(Spp1-EGFP)PD43Gsat/Mmucd (Spp1-EGFP) mouse strains were used. These mice were 6-10 weeks of age.<br>For engraftment of TRAMP-C2 cells, C57BL/6J mice were used. These mice were 6-10 weeks of age.
Wild animals	No wild animals were used in this study.
Reporting on sex	This study does not make any conclusions reporting on sex.
Field-collected samples	No field-collected samples were used in this study..
Ethics oversight	All experimental procedures were approved by the Institutional Animal Care and Use Committee at UCSF.

Note that full information on the approval of the study protocol must also be provided in the manuscript.

Clinical data

Policy information about clinical studies

All manuscripts should comply with the ICMJE guidelines for publication of clinical research and a completed CONSORT checklist must be included with all submissions.

Clinical trial registration	NCT02655822
Study protocol	Details of the clinical protocol were included in a prior report: https://pubmed.ncbi.nlm.nih.gov/31732494/
Data collection	Details on data collection were included in a prior report: https://ascopubs.org/doi/10.1200/JCO.2020.38.6_suppl.129
Outcomes	PSA and RECIST responses were pre-defined secondary endpoint.

Plants

Seed stocks	N/A
Novel plant genotypes	N/A
Authentication	N/A

Flow Cytometry

Plots

Confirm that:

☒ The axis labels state the marker and fluorochrome used (e.g. CD4-FITC).

☒ The axis scales are clearly visible. Include numbers along axes only for bottom left plot of group (a 'group' is an analysis of identical markers).

☒ All plots are contour plots with outliers or pseudocolor plots.

☒ A numerical value for number of cells or percentage (with statistics) is provided.

Methodology

Sample preparation	Organs were harvested and processed as follows: Spleens were mechanically dissociated with FACS wash buffer (FWB: PBS supplemented with 2% (v/v) FBS and 0.5 mM EDTA (Teknova). Tumors were sequentially digested 3 times with 12 ml of a cocktail of 2 mg/ml (w/v) Collagenase Type IV and 100 Kunitz U/ml DNase I (both from Sigma-Aldrich) for 12 minutes per digest. All single-cell suspensions were filtered using 70 µm filters (Fisher Scientific) and subjected to red blood cell lysis using ACK Lysing Buffer (Quality Biological). Cells were immunostained by incubating at 4°C for 30 minutes with fluorescently labeled antibodies below (all antibodies were purchased from BioLegend, unless otherwise indicated). After staining, cells were washed 1-2 times in FWB and resuspended in FWB or FWB containing 1 µg/ml propidium iodide (PI; BioLegend) to assess viability.
Instrument	LSRFortessa X-50, BD FACSAria Fusion flow cytometers
Software	BD FACSDiva for collection, Tree Star FlowJo for analysis
Cell population abundance	For enrichment of mouse CD8+ T cells, single-cell suspensions of spleens from CRPC-bearing mice were labeled with BD Violet Proliferation Dye 450 (Fisher Scientific) and subsequently negatively enriched using the MojoSort™ Mouse CD8 T Cell Isolation Kit, according to the manufacturer's instructions. For isolation of specific myeloid subsets, single-cell suspensions from CRPC developed in Spp1-EGFP mice were incubated with LIVE/DEAD Fixable Dead Cell Stain Kit (Aqua), anti-mouse CD11b-Brilliant Violet 605 (M1/70), CX3CR1-PE/Cyanine7 (SA011F11), F4/80-Alexa Fluor 647 (T45-2342; BD Biosciences), I-A/I-E-Alexa Fluor 700 (M5/114.15.2), Ly-6G-APC/Cyanine7 (1A8), Podoplanin-PerCP/Cyanine5.5 (8.1.1), and Siglec-F-Brilliant Violet 421 (BD Biosciences) antibodies. Following immunostaining, cells were washed twice in FWB and resuspended in FWB containing 1 µg/ml propidium iodide to assess viability. The cells of interest were FACS-purified using BD FACSAria Fusion (BD Biosciences).
Gating strategy	Cells were initially gated based on size (FSC/SSC), followed by size (FSC) to exclude cell doublets. Live cells were then gated based on viability dye before analysis for cellular markers, as shown in the Extended Data Figures.

☒ Tick this box to confirm that a figure exemplifying the gating strategy is provided in the Supplementary Information.
